# Toward Spatial Control of Reaction Selectivity on Photocatalysts Using Area-Selective Atomic Layer Deposition on the Model Dual Site Electrocatalyst Platform

**DOI:** 10.1021/acsnano.4c10387

**Published:** 2024-12-09

**Authors:** W. Wilson McNeary, William D. H. Stinson, Moaz Waqar, Wenjie Zang, Xiaoqing Pan, Daniel V. Esposito, Katherine E. Hurst

**Affiliations:** † Catalytic Carbon Transformation and Scale-Up Center, National Renewable Energy Laboratory, Golden, Colorado 80401, United States; ‡ Department of Chemical Engineering, Columbia Electrochemical Engineering Center, Lenfest Center for Sustainable Energy, Columbia University in the City of New York, New York, New York 10027, United States; § Department of Materials Science and Engineering, University of California Irvine, Irvine, California 92697, United States; ∥ Energy Conversion and Storage Systems Center, National Renewable Energy Laboratory, Golden, Colorado 80401, United States

**Keywords:** area-selective deposition, atomic layer deposition, electrocatalysis, oxide coatings, hydrogen production, scanning electrochemical microscopy, dual-site photocatalyst

## Abstract

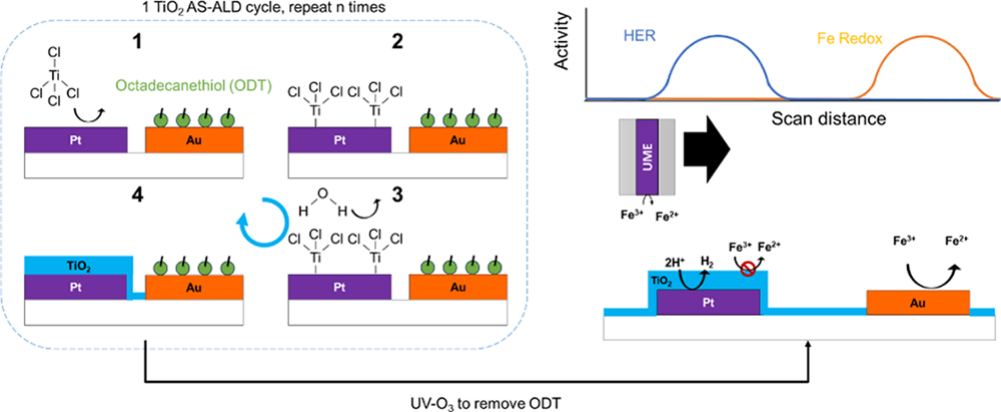

Photocatalytic water splitting is a promising route to low-cost, green H_2_. However, this approach is currently limited in its solar-to-hydrogen conversion efficiency. One major source of efficiency loss is attributed to the high rates of undesired side and back reactions, which are exacerbated by the proximity of neighboring oxidation and reduction sites. Nanoscopic oxide coatings have previously been used to selectively block undesired reactants from reaching active sites; however, a coating encapsulating the entire photocatalyst particle limits activity as it cannot facilitate both half-reactions. In this work, area selective atomic layer deposition (AS-ALD) was used to selectively deposit semipermeable TiO_2_ films onto model metallic cocatalysts for enhancing reaction selectivity while maintaining a high overall activity. Pt and Au were used as exemplary reduction and oxidation cocatalyst sites, respectively, where Au was deactivated toward ALD growth through self-assembled thiol monolayers while TiO_2_ was coated onto Pt sites. Electroanalytical measurements of monometallic thin film electrodes showed that the TiO_2_-encapsulated Pt effectively suppressed undesired H_2_ oxidation and Fe(II)/Fe(III) redox reactions while still permitting the desired hydrogen evolution reaction (HER). A planar model photocatalyst platform containing patterned interdigitated arrays of Au and Pt microelectrodes was further assessed using scanning electrochemical microscopy (SECM), demonstrating the successful use of AS-ALD to enable local reaction selectivity in a dual-reaction-site (photo)electrocatalytic system. Finally, interdigitated microelectrodes having independent potential control were used to show that selectively deposited TiO_2_ coatings can suppress the rate of back reactions on neighboring active sites by an order of magnitude compared with uncoated control samples.

Scalable and cost-effective production of green hydrogen from water electrolysis is expected to play a critical role in decarbonizing many hard-to-abate sectors of the economy.^1,2^ Photocatalytic water splitting provides a promising route to achieving this target due to the potential to directly use incident solar energy to produce hydrogen using low-cost materials and simple reactor design concepts under mild reaction conditions.^3,4^ In addition to resolving durability challenges for photocatalytic materials,^5,6^ a major barrier to the commercialization of this technology is achieving high (>10%) system solar-to-hydrogen conversion (STH) efficiency.

One approach to improve STH efficiency of photocatalytic water splitting systems, while enabling intrinsically safe operation, is to employ the so-called dual-compartment Z-scheme water splitting, whereby two different light absorbing particles—a hydrogen (H_2_) evolving particle (HEP) and an oxygen (O_2_) evolving particle (OEP)—are operated in separate compartments while being electronically coupled by a soluble redox mediator that shuttles charge between them.^7,8^ However, a major challenge with these systems is that the use of redox mediators introduces additional undesired reactions that can severely decrease STH efficiency.^9^ For example, the reduction reaction site on the HEP could catalyze either the hydrogen evolution reaction (HER) or the undesired reduction of the oxidized mediator species (A), as shown in Figure 1a. Unfortunately, the task of selectively carrying out the desired reactions is made more challenging by the greater thermodynamic driving forces for the undesired reactions and facile transport of redox species across the short distances separating neighboring photocatalyst particles (Figure 1b) or reaction sites on the same particle. For dual-component Z-scheme photocatalytic water splitting to achieve high STH efficiency, it is, therefore, crucial to design selective active sites that facilitate only the desired reactions while attenuating undesired reactions.

**Figure 1:**
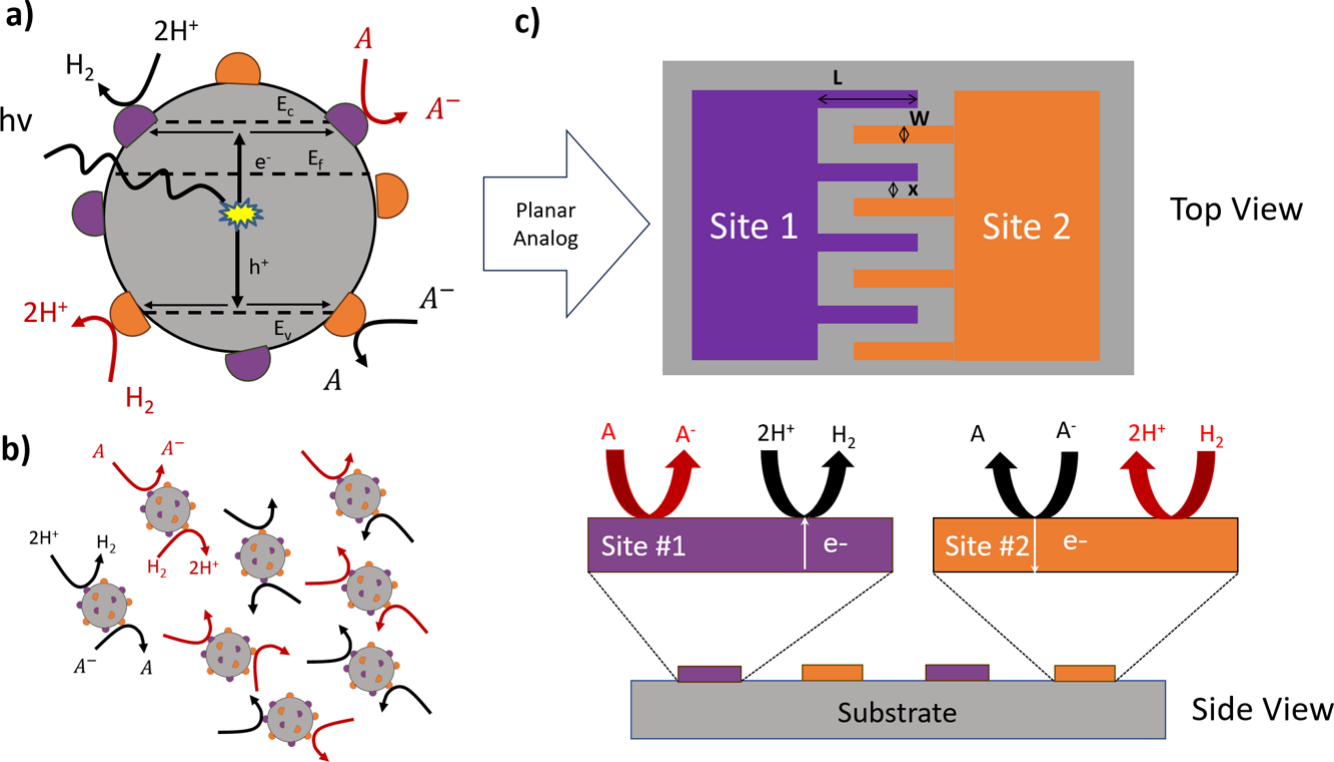
(a) Schematic of an individual hydrogen-evolving particle (HEP) containing two distinct types of cocatalysts (orange and purple hemispheres), each of which can catalyze the desired (black arrows) and undesired reactions (red arrows). (b) Schematic of an ensemble of photocatalytic HEPs in the presence of a redox mediator, where photogenerated product species can be converted back to the reactant(s) by back reactions at neighboring particles or active sites. (c) Schematic top and side-views of a planar photocatalyst analog system comprised interdigitated microelectrodes, with controllable dimensions (*W*: electrode width, *L*: electrode length, *x*: electrode separation distance) that mimic distinct cocatalysts having different potential control.

Nanoscopic overlayers have previously been demonstrated to inhibit the transport of undesired reactants from reaching catalytic active sites while still allowing the desired redox reaction to occur.^10^ Binary oxides such as TiO_*x*_,^11^ CrO_*x*_,^12^ Al_2_O_3_,^13^ and SiO_*x*_^14,15^ have been applied in this way to photocatalytic systems, resulting in improved STH efficiency compared to uncoated controls. However, the best STH efficiencies have still been limited to ∼1% under 1 sun illumination and ambient conditions.^16^ STH efficiency can be increased through temperature,^17^ vacuum,^17^ and pH control,^18^ but designing more selective overlayers may allow for suppressing back reactions at milder conditions. Overlayers for photocatalysts are typically photodeposited selectively onto either the reduction or oxidation reaction sites,^15,16^ as coating the entirety of the particle would likely also block desired reactions from occurring.^19^ However, photodeposition relies on local electrochemical reactivity, which can be difficult to control and lead to nonuniform overlayers with variable performance and morphology. As such, there is a critical need for a controlled and tunable process to selectively deposit overlayers on specific catalytic sites.

Atomic layer deposition (ALD) is a tunable gas phase deposition process that can synthesize nanoscopic coatings onto a wide range of materials,^20^ where deposition conditions (e.g., choice of precursor, deposition temperature) can alter the film properties.^21^ The ALD process can be made spatially selective, known as area selective ALD (AS-ALD), through modification of the substrate surface.^22^ A widely used strategy for protecting the nongrowth area of a surface is the application of self-assembled monolayers, such as 1-octadecanethiol (ODT).^23^ The thiol molecule impedes the adsorption of the ALD precursor, thereby delaying or eliminating nucleation and growth of the ALD film.^23−25^ This approach is often used to block ALD growth on catalyst sites while an ALD coating is deposited on a surrounding surface.^26,27^ Given the range of oxides that can be deposited in this manner, an AS-ALD process holds great utility in the creation of species-selective overlayers for photocatalyst particles.

While AS-ALD has previously been demonstrated on supported nanoparticle catalysts,^27,28^ it can be beneficial to demonstrate the synthesis methodology and benefits of AS-ALD on model platforms containing larger micron-sized features. The AS-ALD process and resultant films can be characterized with more readily accessible microscopy and spatially resolved electrochemistry tools at the micron scale. Similarly, well-defined microelectrodes operating at different electrochemical potentials—much like two different cocatalysts on a photocatalyst particle— allow for more facile characterization of local reaction rates and the extent to which area selective coatings suppress undesired back reactions by scanning electrochemical microscopy (SECM) methods, which becomes exceedingly challenging at the nanoscale in addition to other confounding phenomena.^29,30^ SECM is commonly used to measure local reactivity of surfaces with spatial resolution.^31^ and has previously been used to measure partial currents from two competing reactions.^32,33^ Thus, SECM can be used to evaluate differences in the electrochemical properties of patterned surfaces.^34,35^ Herein, we employ SECM to study patterned samples based on interdigitated band electrodes (Figure 1c), which serve as planar analogs to photocatalyst particles. In this design, the microelectrode arrays represent two different cocatalyst materials whose size, composition, and separation distance can be varied. This platform furthermore allows for independent control and/or measurement of the potentials of the two different microelectrode arrays, which can be used to mimic the different potentials achieved at the oxidation and reduction sites on an illuminated photocatalyst particle.

The remainder of the study is organized as follows. First, a TiO_2_ AS-ALD methodology is described and demonstrated using monometallic (Au, Pt) thin film electrodes. The deposition of TiO_2_ was pursued due to its previous use as a photocatalyst coating,^11,36^ inherent tunability of the material in terms of ionic and electrical conductivities, and relative ease of the ALD chemistry (e.g., mild temperatures, reactive precursors with high vapor pressures). Next, we show that the same AS-ALD methodology can be successfully applied to “dual-site” samples based on interdigitated arrays of Pt and Au band electrodes (Figure 1c). After confirming the selective deposition of TiO_2_ overlayers on Pt by spectroscopy and microscopy, SECM measurements of the local reactivity of the HER and Fe redox reactions on the TiO_2_ | Pt and Au bands are presented, demonstrating the effectiveness of AS-ALD for enhancing reaction selectivity. Finally, the rates of undesired back reactions measured for interdigitated electrodes deposited on a nonconductive substrate are reported, revealing a 10-fold decrease in the back reaction rate for AS-ALD samples compared to unencapsulated controls.

## Results and Discussion

### Blocking ALD Growth on Monometallic Electrodes Using ODT

**Figure 2:**
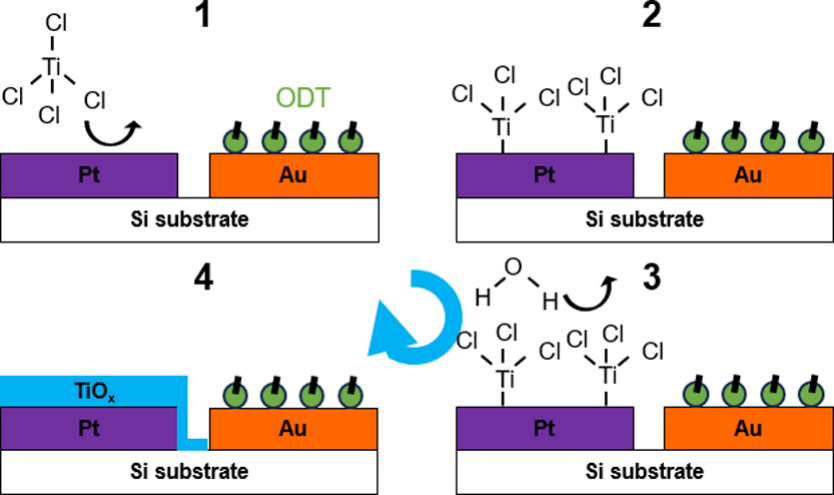
AS-ALD growth approach for selective deposition of TiO_2_ onto Pt sites, with the two half cycles of TiCl_4_ and H_2_O depicted in four steps.

For a dual-site hydrogen evolving particle, it is desired to selectively deposit a semipermeable oxide overlayer on the reduction reaction site so as to block the redox mediator reactions. Platinum, a typical HER catalyst,^37^ was used as the surrogate reduction catalyst while gold was used as an oxidative catalyst. Au exhibits strong adsorption of ODT to create self-assembled monolayers, which are known to inhibit ALD growth.^23^ Au substrates were soaked overnight in a 50 mM solution of ODT and then exposed to alternating pulses of TiCl_4_ and H_2_O in a custom-built ALD reactor at 150 °C. This approach is shown in Figure 2, where the ODT blocks the adsorption of the precursor molecules, inhibiting the growth of the TiO_2_. Substrates were exposed to various numbers of cycles to achieve films with different thicknesses.

**Figure 3:**
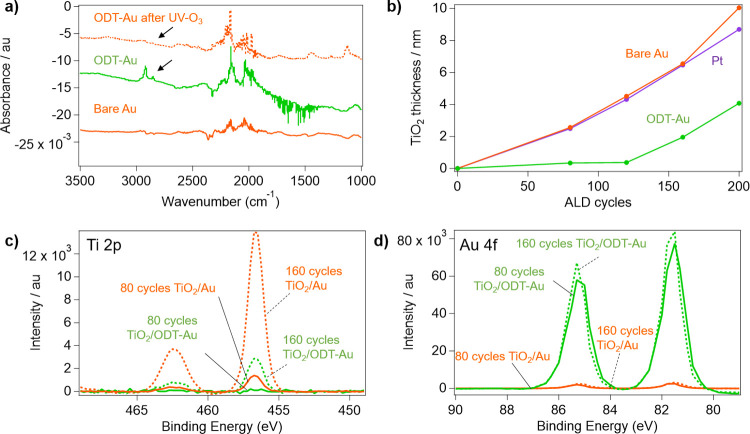
(a) FTIR spectra of monometallic Au, ODT-Au, and UV–O_3_-treated ODT-Au confirm the appearance and disappearance of C–H stretches (black arrow) relative to the unmodified surface. (b) TiO_2_ thickness as determined by ellipsometry on monometallic Pt, Au, and ODT-Au surfaces at varying numbers of ALD cycles. XPS (c) Ti 2p and (d) Au 4f spectra of Au (orange trace) and ODT-Au (green trace) surfaces subjected to 80 (solid trace) and 160 (dotted trace) ALD cycles.

Initially, the TiO_2_ ALD process was performed on individual monometallic samples comprised of 50 nm thin films of Pt, Au, or Au modified with ODT (ODT-Au). All monometallic thin film catalysts were deposited via an electron beam onto degenerately doped Si wafer substrates. Adsorption of the ODT layer on the Au electrode was confirmed by FTIR (Figure 3a), as evidenced by the appearance of significant C–H stretching peaks in the range of 3000–2800 nm^–1^. Thermal annealing of the ODT-Au electrode in He up to the ALD temperature of 150 °C (Figure S1) was found to reduce the intensity of these stretching peaks but did not completely remove them, implying that the ODT layer should be retained in the ALD reaction environment. Previous work^23^ has also demonstrated the stability of ODT layers bound to metallic substrates at temperatures ≥150 °C. A subsequent ultraviolet ozone (UV–O_3_) treatment was found to remove the ODT as evidenced by the disappearance of those same C–H stretching peaks. Removal of residual ODT by UV–O_3_ was necessary to ensure that the electrochemically active surface was free of contaminants. As such, this UV–O_3_ procedure was adopted as a post-ALD synthesis cleaning procedure that was applied to all samples before electrochemical, X-ray photoelectron spectroscopy (XPS), and scanning transmission electron microscopy (STEM) analyses. Ellipsometry was conducted on the ALD-coated samples before UV–O_3_ treatment to monitor the selective growth of TiO_2_ (Figure 3b). The TiO_2_ thickness increased linearly on unmodified Au and Pt from the first cycle, reaching about 9 nm after 200 cycles. In sharp contrast, a nucleation delay occurred on the ODT-Au surface, with the growth of TiO_2_ barely detected until after 120 cycles. Beyond this point, the growth advanced with a similar linearity as on the unmodified substrates.

These findings were corroborated by XPS on the UV–O_3_-treated surfaces (Figure 3c,d), which showed little to no signal in the Ti 2p region for the 80-cycle ODT-Au electrode, while a moderate Ti 2p signal was observed after 160 cycles. The Au 4f signal remained high at both 80 and 160 cycles on ODT-Au, indicating that the underlying Au surface remained highly exposed, even beyond the nucleation delay. This suggests that any TiO_2_ nucleation and growth on ODT-Au up to this point were likely present as nanoislands rather than a continuous film. It is also notable that the Ti 2p peak centers shift to higher binding energies with an increasing number of ALD cycles. Despite the use of a charge neutralizer during XPS measurements, this observation is most likely attributed to differences in surface charging arising from the growing thickness of the TiO_2_ overlayer. Additionally, the ratio of the O 1s signal associated with lattice oxygen (Ti–O–Ti) to Ti 2p was found to be nearly stoichiometric with values ranging between 1.78 and 1.88 for all non-ODT modified samples (Figures S2, S3, and Table S1); as such, the nomenclature of TiO_2_ is used for oxide deposition in this work. The XPS and ellipsometry results are consistent with the fact that ODT acts as an effective blocking agent preventing TiO_2_ growth up to 160 cycles. However, some submonolayer TiO_2_ nucleation and growth were detected on the inhibited surface beyond 160 cycles (indicating the end of the nucleation delay) and would be expected to continue with additional cycles.

### Electrochemical Behavior of Monometallic TiO_2_/M (M = Au, Pt) Electrodes

**Figure 4:**
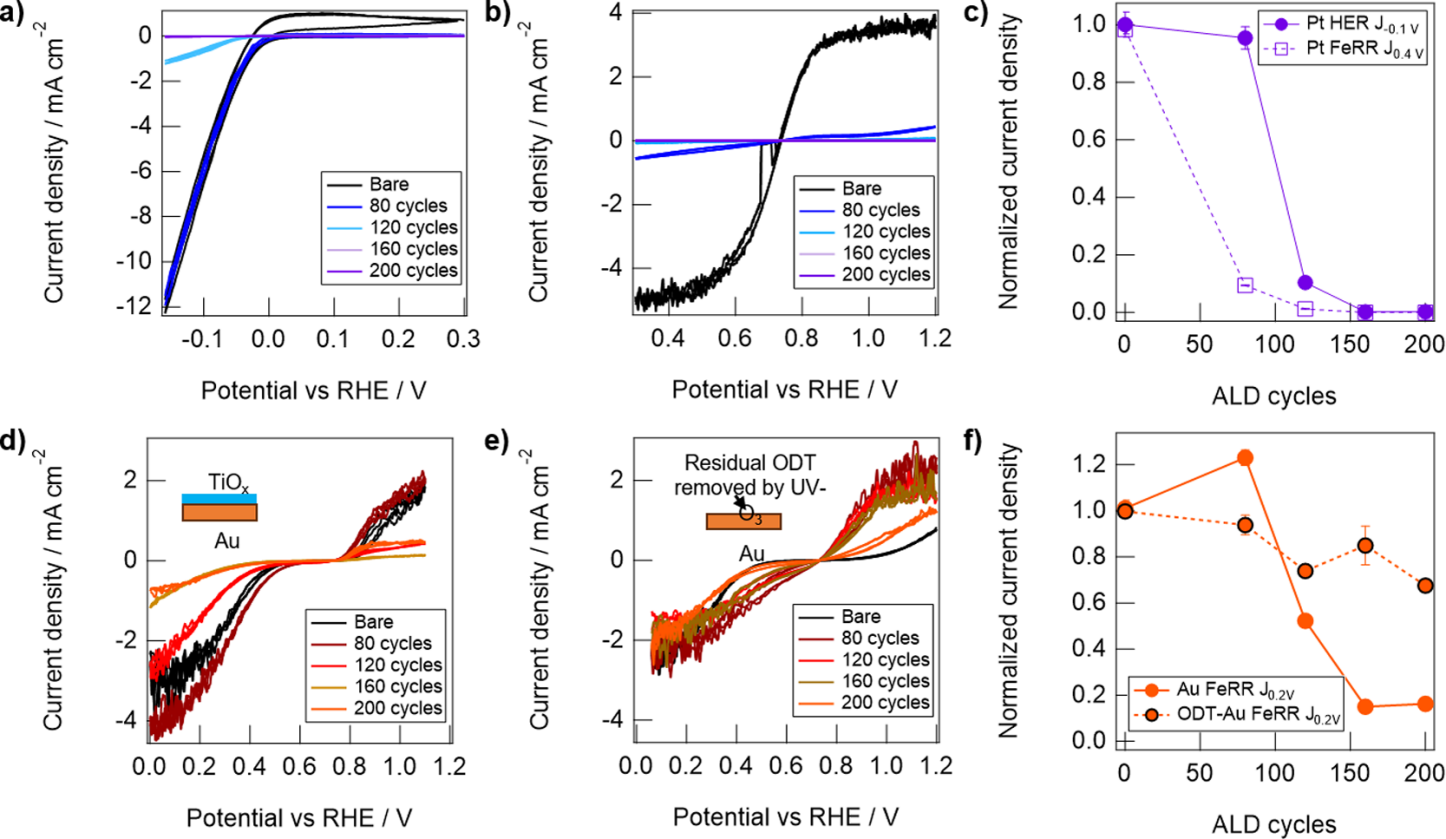
Representative cyclic voltammograms (CV) of Pt electrodes with varying TiO_2_ ALD cycles tested in (a) supporting electrolyte (50 mM H_2_SO_4_+ 100 mM Na_2_SO_4_, pH 1.5) and (b) Fe-containing electrolyte (25 mM FeSO_4_ + 12.5 mM Fe_3_(SO_4_)_2_ + supporting electrolyte), taken at 10 mV s^–1^. (c) Current densities, normalized versus the bare control, of TiO_2_-modified Pt samples toward the HER and FeRR reaction, taken at −0.1 and 0.4 V vs RHE respectively. Representative CVs of (d) Au electrodes and (e) ODT-Au electrodes with varying cycles of TiO_2_ in an Fe-containing electrolyte. (f) Current densities, normalized versus the bare control, of TiO_2_ modified Au and ODT-Au samples toward the FeRR reaction, taken at 0.2 V vs RHE. Error bars in (c) and (f) represent the standard deviation of current densities recorded at the indicated potential (±3 mV) during 3 CV measurements.

To characterize the effect of TiO_2_ overlayers on reaction selectivity, monometallic electrodes (Au, Au-ODT, or Pt) exposed to varying numbers of ALD cycles were tested for activity toward the hydrogen evolution (HER) and Fe(II)/Fe(III) redox reactions. The latter redox couple was chosen due to its common use as a redox mediator in Z-scheme water splitting.^38^ Cyclic voltammetry (CV) experiments were run in the deaerated supporting electrolyte, composed of 50 mM H_2_SO_4_ + 100 mM Na_2_SO_4_, pH 1.5) (Figure 4a), and Fe-containing electrolyte composed of supporting electrolyte with an additional 25 mM FeSO_4_ + 12.5 mM Fe_2_(SO_4_)_3_ (Figure 4b, d, e).

Electrochemical data in Figure 4a show the primary feature in all supporting electrolyte scans for TiO_2_/Pt electrodes was associated with the HER, which occurs at potentials more negative than 0.0 V vs RHE for Pt electrodes. The HER activity of the Pt electrode was substantially decreased with the deposition of 120 cycles of TiO_2_ or more, indicating that TiO_2_ thickness should be kept below 4 nm (based on Figure 3c) to avoid significant inhibition of the desired HER. Figure 4b shows facile kinetics toward Fe redox reactions on the bare Pt electrode, as evidenced by CV curves that pass through the standard reduction potential for the Fe(II)/Fe(III) reaction (*E*_0,Fe_, + 0.77 V vs RHE). At larger overpotentials (>150 mV), mass transport limiting current densities of ∼4.7 mA cm^–2^ are observed. However, 80 cycles (i.e., around 2 nm) of TiO_2_ resulted in a significant suppression in Fe redox current to ∼0.4 mA cm^–2^ (>80% decrease), with samples based on 160 or 200 ALD cycles displaying ∼0.0005 mA cm^–2^ (>99% decrease). Figure 4c displays the normalized decrease in the HER and FeRR currents for the Pt electrodes as a function of the number of TiO_2_ ALD cycles, revealing that thicker TiO_2_ overlayers are required to decrease the HER signal compared to the FeRR signal. This observation can most likely be attributed to a higher permeability of H^+^ through TiO_2_ than Fe(III), which is well-supported by theory. Molecular dynamics simulations have shown that H^+^ transport within confined TiO_2_ nanopores is aided by facilitated diffusion along the TiO_2_ surface,^39^ while Fe(II) and Fe(III) diffusion through oxide nanopores is greatly inhibited by the large energetic barrier associated with dehydration and distortion of water molecules from the second and third ion solvation shells.^40^ As such, we surmise that most of the Fe redox reaction likely occurs at the outer surface of thinner TiO_2_ overlayers, which becomes limited by high electrical resistance at higher overlayer thicknesses.^41^

Au electrodes were similarly tested in both the supporting and Fe-containing electrolytes. Au, being a less active HER catalyst than Pt,^42^ required an additional ∼200 mV of overpotential for the onset of the HER. Thus, minimal HER current is seen for all Au electrodes in supporting electrolytes over the potential range investigated (Figure S5). Figure 4d displays representative CV curves for Au electrodes in a Fe-containing electrolyte. Unlike Pt, the CV curves for bare Au exhibit significant asymmetry in the Fe redox features, with faster kinetics toward the Fe(II) oxidation reaction (FeOR) than the Fe(III) reduction reaction (FeRR). This could be due to the beneficial adsorption of SO_4_^–^, only seen in the supporting electrolyte CV scans above 0.7 V vs RHE (Figure S5). A larger range of potentials was scanned for these Au electrodes to observe mass-transport limiting behavior at larger overpotentials for the Fe redox reactions. Significant decreases in current density were observed for TiO_2_ overlayers of 120 cycles (i.e., around 4 nm) or more, from ∼3 mA cm^–2^ for bare Au to 0.05 mA cm^–2^ for 200 cycles. A comparable trend in normalized FeRR current is seen for the Au (Figure 4f) and Pt electrodes (Figure 4c), suggesting a similar effect of the TiO_2_ overlayer on suppressing the FeRR signal.

In contrast to unmodified Au, ODT-Au substrates (Figure 4e), showed significant currents toward the Fe redox reactions, reaching almost identical limiting currents for both the FeRR and FeOR. Normalized FeRR current densities, taken at 0.2 V vs RHE, show only a 30% suppression in current for the ODT-Au electrode exposed to 200 ALD cycles while the unprotected Au electrode showed a > 80% decrease in FeRR signal when coated with the same number of cycles (Figure 4f). This behavior is consistent with ellipsometry measurements (Figure 2c) in that it indicates the treatment with ODT inhibits the growth of TiO_2_, and subsequently leaves Au reactive sites available for Fe redox reactions. The slight decrease in normalized current density for the ODT-Au FeRR with increasing cycle numbers in Figure 4f may suggest that some amount of electrochemically active surface area is lost to the deposition of TiO_2_ nanoislands at large cycle numbers. In summary, there exists a window of TiO_2_ cycle numbers (80–160 cycles) for which the AS-ALD nucleation delay overlaps with the desired selective reactivity on Pt (Figure 3c). In this range of TiO_2_ thickness (2–6 nm on Pt), (i) the Fe redox reactions are inhibited on Pt while still permitting the HER to occur and (ii) complete coverage of TiO_2_ on Au is avoided, allowing the exposed Auto still facilitate Fe oxidation, as desired for a HEP.

### Planar Photocatalyst Analog Based on Interdigitated Band Electrodes

A planar model was developed using an interdigitated electrode composed of metal features (Figure 1c) that represent oxidation and reduction reaction sites of a photocatalyst particle. While the length scales separating neighboring microelectrodes (100 μm) on this model platform are still magnitudes larger than the typical interparticle (0.01–1 μm) and cocatalyst (<1–100 nm) separation distances in a dual-site photocatalytic particle, the ability to systematically vary cocatalyst geometry and composition, as well as the relative ease of characterizing the microelectrodes, make this a useful platform to study the influence of overlayers on the behavior of neighboring “co-catalysts”. As the study focused on electrochemical reactions relevant to an HEP, 160 cycles of TiO_2_ were selectively deposited onto the Pt bands of the interdigitated Au and Pt electrode analog.

**Figure 5:**
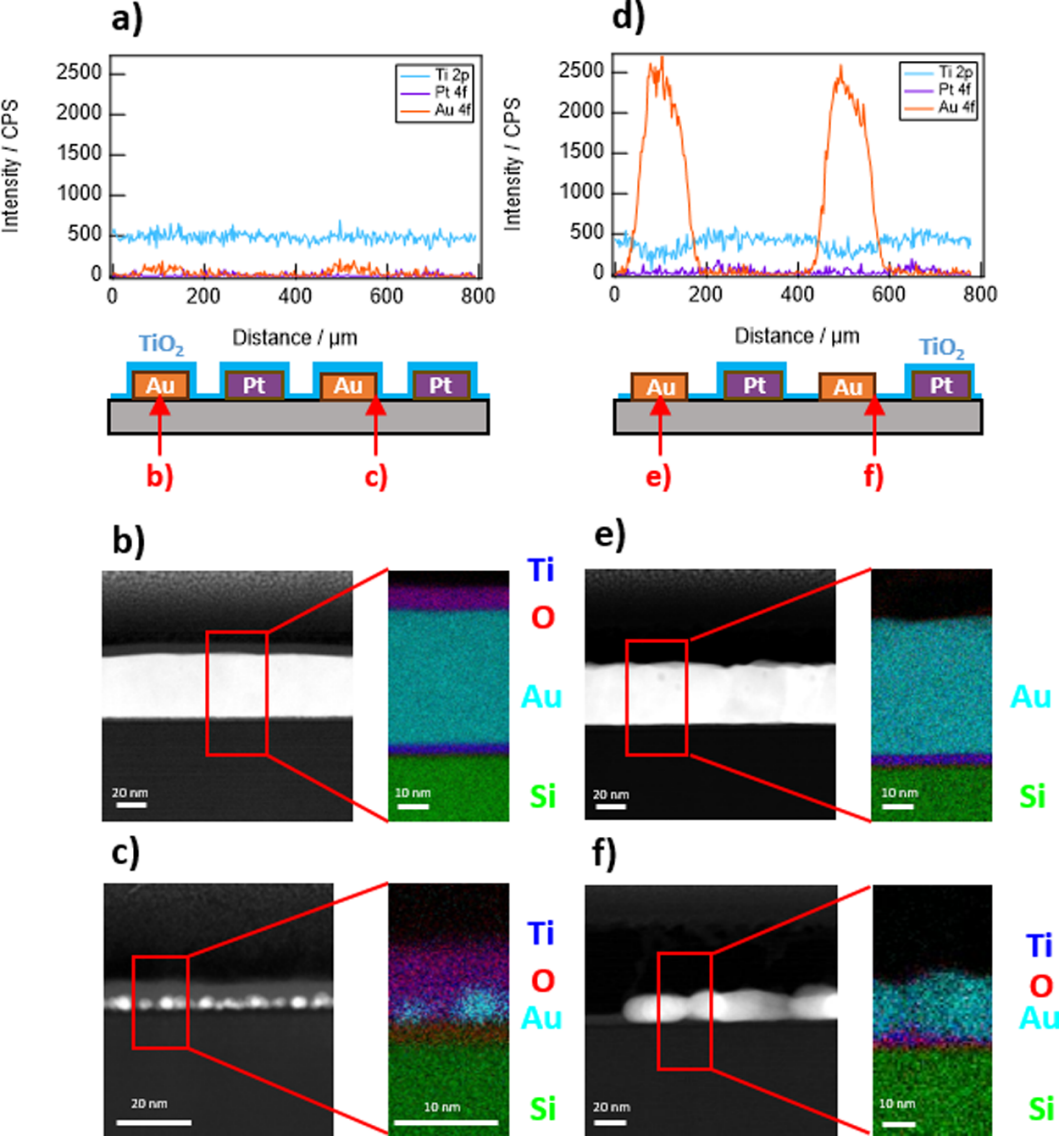
XPS line scans measured perpendicular to interdigitated Au and Pt band electrodes for (a) 160 cycles TiO_2_ on the Pt | Au electrode and d) 160 cycles TiO_2_ on the Pt | ODT-Au electrode. (b,c) and (e,f) Representative STEM/EDS images of the center and edge locations of Au band electrodes present in the same samples used in the XPS line scans provided in (a) and (d), respectively. Schematics of sample cross-sections provided below (a) and (d) are aligned with the corresponding band locations shown in (a) and (d) and also contain red arrows to indicate the locations of TEM/EDS measurements in (b), (c), (e), and f).

A combination of microfocused (≈ 10 μm) scanning XPS and cross-sectional STEM, with energy dispersive X-ray spectroscopy (EDS), were used to characterize the locations of TiO_2_ deposition on the interdigitated arrays. XPS line scans were taken perpendicular to the length of the interdigitated bands of unmodified Pt | Au (Figures 5a and S6a) and Pt | ODT-Au (Figures 5d, S6e, and S7) electrodes, where both were exposed to 160 cycles of TiO_2_ ALD. Similar to monometallic samples Blocking ALD Growth on Monometallic Electrodes Using ODT, Figure 5a shows that the traditional ALD process lacking the ODT blocking step resulted in a large Ti 2p signal associated with the presence of the TiO_2_ overlayer over the entire line scan, while negligible Au 4f and Pt 4f signal is observed due to screening by the TiO_2_ overlayer. This result is supported by STEM/EDS measurements, which showed that a conformal TiO_2_ overlayer of approximately 6 nm thickness was deposited over both the center (Figure 5b) and the edge (Figure 5c) of the band electrodes. Additional STEM images of the Pt areas of both Pt | Au and Pt | ODT-Au, shown in Figure S6, confirm a similar deposition over the Pt area. In both of these samples, the TiO_2_ overlayer was amorphous (as evidenced by the lack of crystalline order in the high-magnification images of Figure S6d,h), which is expected for TiCl_4_–H_2_O ALD chemistry at growth temperatures ≤150 °C.^43^ These images display a similar thickness of TiO_2_ overlayers (5–6 nm) as measured by ellipsometry of monometallic Au thin film electrodes (Figure 3c). Additional XPS area scans show a uniform Ti 2p signal over a larger area (Figure S8), suggesting uniform deposition as expected of ALD.

In contrast, a large Au 4f signal was observed from the AS-ALD sample (Figure 5d) with a simultaneous decrease in the Ti 2p signal over the Au bands, suggesting minimal growth of TiO_2_ on Au. This is consistent with STEM/EDS measurements taken at the center of Au bands on the Pt | ODT-Au sample (Figure 5e), which did not show any evidence of deposited TiO_2_ in these regions. The edges of Au bands on Pt | ODT-Au (Figures 5f, S4, and S7) showed a slight presence of Ti and O species, which may be indicative of small nanoislands of TiO_2_ that did not coalesce into a uniform film. These islands were likely responsible for the nonzero Ti 2p signal over the Au regions in Figures 5d and 3d. The near absence of the Pt 4f signal in Figure 5a,d also indicates the growth of the TiO_2_ over the Pt features. Thus, at 160 cycles of TiO_2_, the ODT was effective at preventing full monolayer ALD growth on the Au bands of the Pt | ODT-Au interdigitated electrodes.

**Figure 6:**
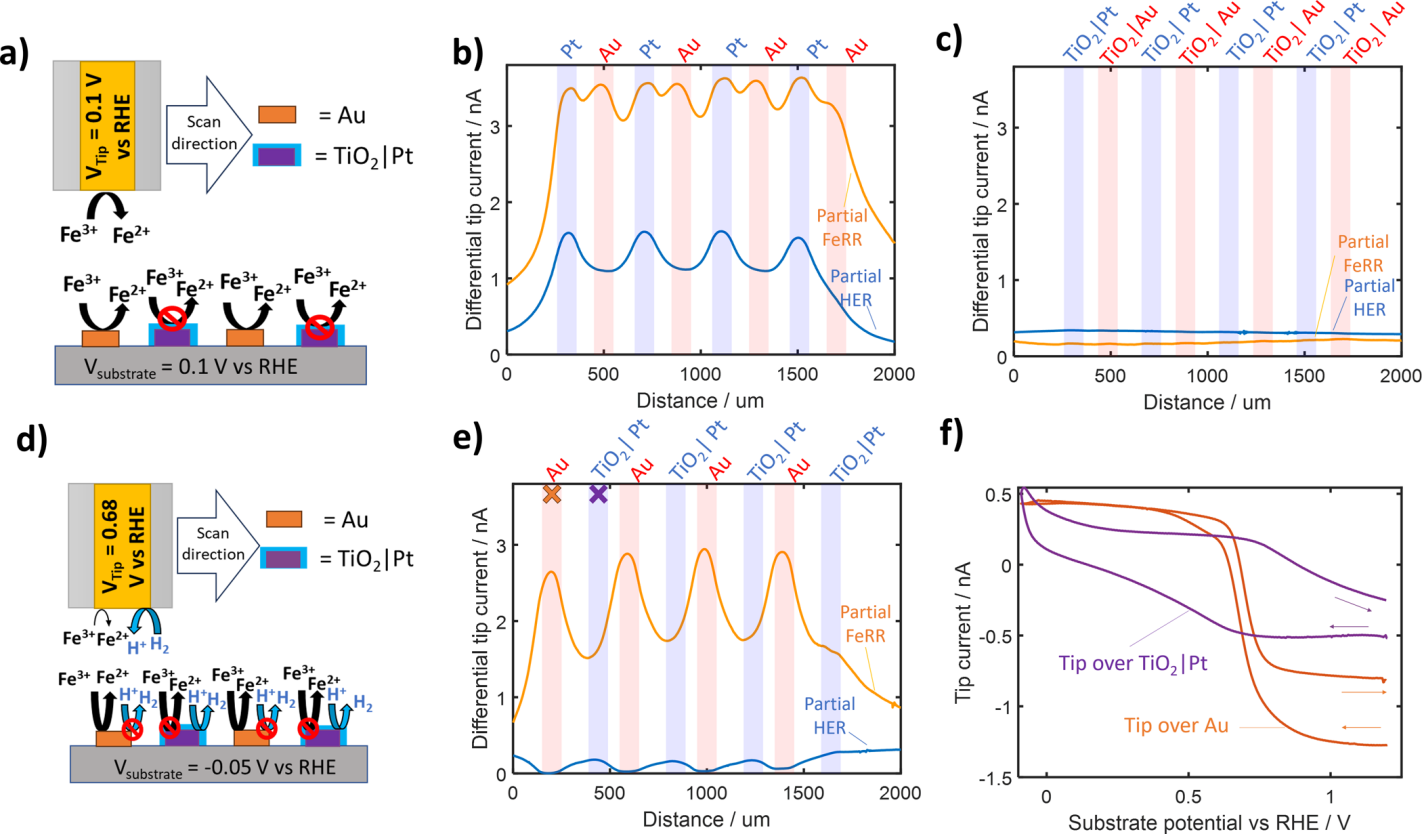
Schematic of SECM sensing methodology where the substrate potential was set to either (a) 0.1 V vs RHE, while the tip is held at 0.1 V for FeRR estimation (competition mode) or (d) −0.05 V vs RHE while the tip is held at 0.8 V vs RHE for HER estimation (SGTC mode). Estimated partial currents for the FeRR (difference between 0.1 V, and OCP scans) and the HER (difference between −0.05 and 0.1 V scans) of (b) bare, (c) a 160 cycle ALD coated, and (e) a 160 cycle AS-ALD-coated Au | Pt-interdigitated electrode. (f) Tip current measured with a tip potential of 0.68 V vs RHE while located over AS-ALD sample at marked (X) locations in (e) while the substrate was cycled between −0.1 and 1.2 V vs RHE at 20 mV s^–1^, with the scan direction signified by small arrows. Additional details on the SECM methodology, including all raw data sets, can be seen in ESI Section VI.

To confirm that the interdigitated band electrodes have similar electrochemical behavior to monometallic electrodes, scanning electrochemical microscopy (SECM) was used to locally measure the activity toward the HER and FeRR over both Pt and Au band electrodes under identical conditions. SECM is a scanning probe microscopy that uses an ultramicroelectrode (UME) tip placed close to a surface to electrochemically sense redox species generated on the sample surface in the local vicinity of the UME probe.^31^ The substrate and UME potentials can be varied relative to standard reduction potentials of redox species present in the electrolyte to allow the SECM measurement to selectively detect one or more redox species, thereby allowing the user to image local reaction rates for more than one reaction.^32,33^ To estimate the relative amount of FeRR current over each band electrode, SECM line scans were completed using competition mode with the substrate potentials set to either 0.1 V vs RHE (where the substrate drives only FeRR) or at open circuit (OCP, where the substrate has minimal current) while keeping the tip potential fixed at 0.1 V vs RHE, where the UME current is proportional to the Fe(III) concentration (Figure 6a). Similarly, local differences in substrate HER activity were estimated using the substrate-generation tip-collection (SGTC) mode by holding the substrate at −0.05 V vs RHE (for which it is possible to catalyze both HER and FeRR) or 0.1 V vs RHE (for which only FeRR can occur) while fixing the tip potential at 0.68 V vs RHE. This tip potential was chosen because it is the reversible potential for Fe(II)/Fe(III) redox seen in tip CV curves in the Fe electrolyte (Figure S9), which allows the UME to oxidize H_2_ with minimal interference from Fe(II) or Fe(III) redox reactions. By taking the difference in tip current recorded under the two different conditions described above, the partial currents for each reaction can be estimated.^33^ Additional details on the SECM methodology can be found in ESI Section SVI.

The SECM measurements were completed in Fe-containing electrolyte (1.5 mM Fe_2_(SO_4_)_3_+ 3 mM FeSO_4_ + 100 mM Na_2_SO_4_ + 50 mM H_2_SO_4_) on three different interdigitated band electrodes: uncoated, ALD, and AS-ALD interdigitated electrodes. The ALD and AS-ALD interdigitated electrodes shown in Figure 6 were exposed to 160 cycles of TiO_2_ ALD. Results obtained for samples made with 80 and 120 AS-ALD cycles are additionally provided in ESI Section SX. The CVs of the interdigitated electrodes (Figure S11) show pronounced Fe(II)/Fe(III) redox features for the uncoated Au | Pt electrode, while the ALD electrode exhibited significant suppression of Fe(II)/Fe(III) features, as expected for a fully coated sample. The AS-ALD electrode also showed significant Fe(II)/Fe(III) redox peaks, although it is notable that they were highly asymmetric, as had been seen for monometallic bare Au electrodes (Figure 4e).

SECM line scans recorded at each potential are shown in Figure S10. These results were then used to estimate the local partial currents toward the HER and the FeRR over each individual band electrode for an uncoated sample (Figure 6b), an ALD-coated sample (Figure 6c), and an AS-ALD sample (Figure 6d). As expected, a large FeRR current was seen over both the Au and Pt bands on the uncoated sample. When the surface was subjected to the ALD process, the FeRR current became negligible over the entire sample, in agreement with the behavior seen for the TiO_2_-coated Au and Pt monometallic electrodes (Figure 4). In contrast, the SECM line scan for the AS-ALD sample (Figure 6e) displayed a significant FeRR signal with maximum and minima centered over the Au and Pt bands, respectively. This observation is consistent with characterization measurements showing that TiO_2_ was selectively deposited onto the Pt bands. A small increase in FeRR current was measured over the last TiO_2_ | Pt feature (located at ∼1800 μm), which resulted from a nearby defect in the overlayer (Figure S12). It is also notable that the peak FeRR currents measured for the AS-ALD sample in Figure 6e are ∼20% lower than those measured for the bare sample in Figure 6b, which can be explained by the fact that the signal measured over Au bands in the uncoated electrode is increased due to diffusion of redox species at the neighboring exposed Pt features. This behavior between neighboring band electrodes is further explored in the next section.

Similar to monometallic electrodes in Section 2.2 (Figure 4a), the only significant HER current was located over the Pt locations for the bare Au | Pt interdigitated electrode, as the Au features are not active toward the HER at this potential (Figure S5). Minimal features were seen in the HER signal over the entire ALD electrode, suggesting a large HER suppression similar to that seen in Figure 4a. Some features were seen in the HER signal over the Pt regions on the AS-ALD electrode but could have originated from additional FeRR occurring on the TiO_2_ | Si areas, as seen in the higher baseline signal at the start and end of the line scan. Static tip measurements based on holding the UME tip directly over a single feature (locations marked with “X” on the schematic in Figure 6e) were used to additionally probe the HER activity over the TiO_2_ | Pt locations. Such measurements allow for higher sensitivity to the reversible H_2_ electrochemistry compared to scanning UME line scan measurements attributed to a decreased influence of lateral diffusion from the neighboring reaction sites that results from the shorter time constants associated with the concomitant substrate CV cycling measurements. For these experiments, the tip was held at 0.68 V versus RHE while the substrate was scanned between −0.1 and 1.2 V versus RHE at 20 mV s^–1^. In Figure 6e, when the tip is located over the Au band, a sharp change in the tip current from negative to positive was seen when the substrate potential became more negative than ∼0.7 V vs RHE, which can be explained by the rapid switch from FeOR to FeRR at the underlying exposed Au band. In contrast, when the tip is over the TiO_2_ | Pt band, the tip current has a slower response time for potentials around 0.7 V vs RHE. This suggests the tip signal is more likely due to the diffusion of Fe(II) species generated at the neighboring Au features, which would have a longer diffusional distance and thus slower response time. A significant oxidation current is additionally seen for potentials below 0 V vs RHE, while no feature was seen over the Au band. This UME oxidation signal at negative substrate potentials can be explained as originating from H_2_ generation at the TiO_2_ | Pt band and subsequent H_2_ oxidation at the UME. This suggests that the TiO_2_ | Pt band is still active toward the HER, but the tip response is low due to the lower substrate currents under the line scan conditions. Additional line scans and CVs were completed in supporting electrolytes, which additionally show a similar HER activity over only the TiO_2_ | Pt features (Figure S13).

Samples of 80 and 120 cycles of the TiO_2_ AS-ALD electrode were also tested and compared to the behavior observed for monometallic samples discussed in Section 2.2. While there were Fe redox features seen over the TiO_2_ | Pt locations for both samples, the HER signal was significantly higher as well (Figure S14). This may suggest that there are minor differences between the monometallic and interdigitated electrodes, which could be related to either thickness variations between the different types of samples, or alternately, the SECM sensing method could be more sensitive than the monometallic CV measurements due to the extended CA conditions in SECM line scans which could generate detectible concentration gradients even for the minor activities seen for TiO_2_|Pt electrodes.

### Simulated Ensemble Measurements

**Figure 7:**
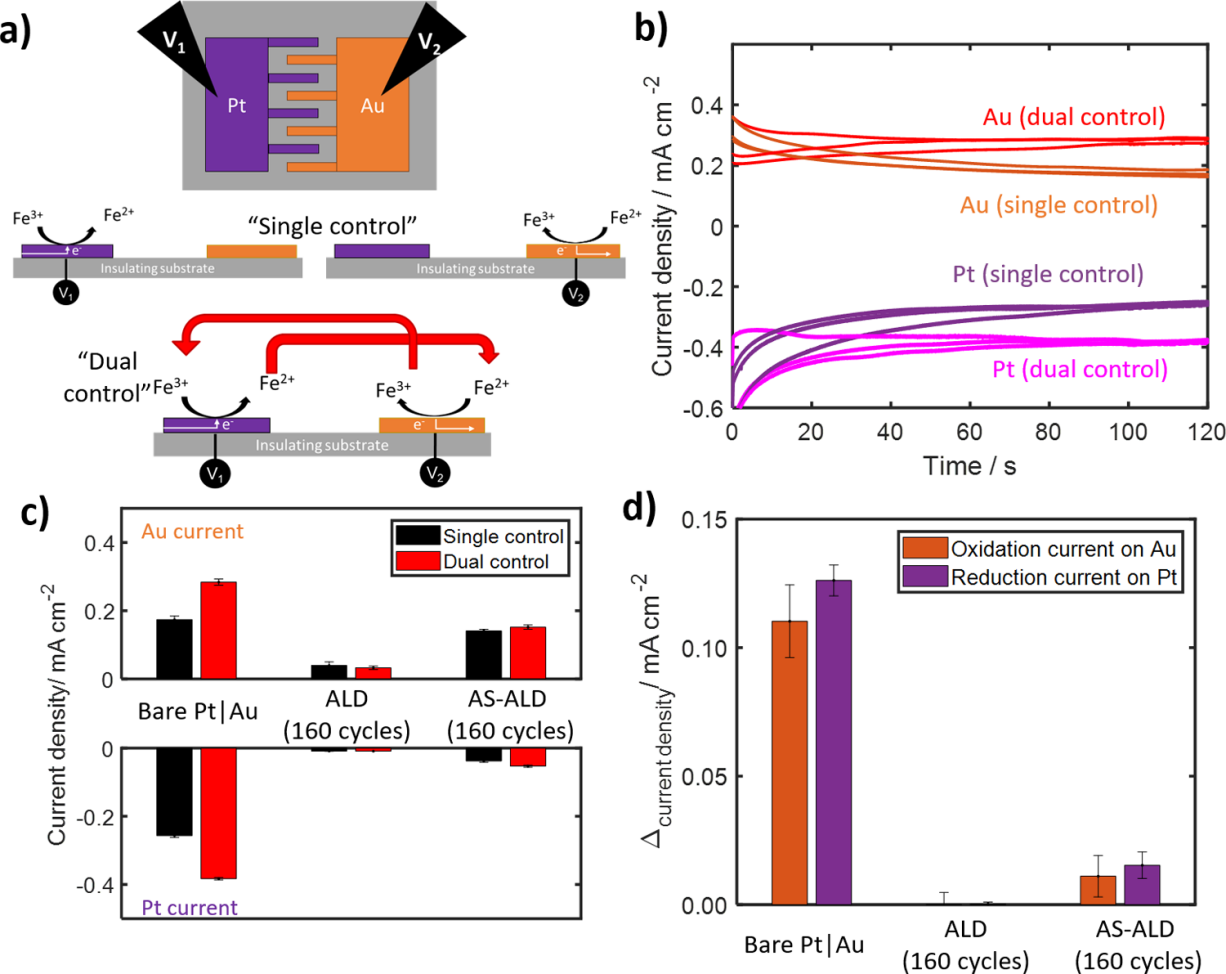
(a) Schematic top and side-views of the interdigitated electrode arrays deposited onto an insulating substrate to allow for independent control of the electrochemical potentials of the Pt (*V*_1_) and Au (*V*_2_) microelectrode arrays. (b) Representative chronoamperometry measurements of bare (uncoated) samples recorded while only controlling the potential of one of the electrode arrays (“single control”, Au at 1.2 V vs RHE, Pt at 0.1 V vs RHE while the other is at open circuit) or while simultaneously controlling the potential of both electrode arrays (“dual control”, Au at 1.2 V vs RHE, Pt at 0.1 V vs RHE). Measurements were completed in 3 mM FeSO_4_ + 1.5 mM Fe_2_(SO_4_)_3_ + 50 mM H_2_SO_4_ + 100 mM Na_2_SO_4_ (pH 1.5). Three different samples were tested with either no ALD (bare), 160 cycles of TiO_2_ ALD, or 160 cycles of TiO_2_ AS-ALD. (c) Currents for both Au and Pt taken at 120 s during CA measurements, averaged over 3 experiments, for both single control (black bars) and dual control (red bars). (d) The difference in current density for the Pt and Au electrode arrays when recorded in “dual control” compared to “single control”, which is used here as a measure of the positive feedback resulting from back reactions. Error bars on (c, d) represent the standard deviation, in the measured current from three separate measurements, with error propagated in (d).

In a photocatalytic particle containing two different types of reaction sites—one for oxidation and one for reduction—it can be expected that the electrochemical potentials of each site will be different when the photocatalyst particle is illuminated.^44^ An example of this behavior is illustrated in Figure 1a, where the reduction sites (purple hemispheres) receiving photogenerated electrons have a potential similar to (depending on charge separation and kinetic phenomena)^37,45^ the semiconductor conduction band (*E*_c_) while the oxidation sites (orange hemispheres) receiving photogenerated holes have a potential similar to the valence band (*E*_v_). Having a potential difference between the two different active sites is necessary to drive electrolytic electrochemical reactions like water splitting but also leads to large overpotentials favoring undesired competing reactions to occur at each site. To emulate the varied potentials at different cocatalyst sites in real photocatalysts, the interdigitated band electrode pattern was deposited onto an insulating glass substrate, which allows for simultaneous and independent potential control of each microband electrode array by using a bipotentiostat connected to each of the two top contact pads shown in Figures 1c and 7a. By applying a more positive electrochemical potential to the Au contact and a more negative reduction potential to the Pt contact, the setup can simulate how active sites operating with different potentials on the same or neighboring photocatalytic particles interact with each other through the diffusive exchange of redox species and subsequently enhanced back reaction rates.

As this study is primarily focused on a HEP analogue, the primary back reactions of interest are the FeRR on the Pt features (reduction site) and the HOR on the Au electrode (oxidation site). To measure the rates of these back-reactions, experiments were carried out in two different electrolytes: (i) a Fe(II)/Fe(III)-containing electrolyte, which was used to measure the rate of undesired FeRR on the Pt band electrodes, and (ii) an Fe(II)/Fe(III)-free electrolyte, which was used to measure the rate of undesired HOR on Au. In both electrolytes, chronoamperometry scans were first recorded with the potential of one microelectrode array fixed (“single control”) while the other microelectrode array was kept at open circuit potential, followed by experiments for which the potentials of both microelectrode arrays were fixed (“dual control”). To study the FeRR back reaction rates under dual potential control in the Fe(II)/Fe(III) containing electrolyte, the Pt and Au potentials were held at *V*_1_ = 0.1 V and *V*_2_ = 1.2 V vs RHE, respectively. Based on the CV curves in Figure S11, both FeOR on Au and FeRR on Pt are expected to be mass transfer limivted at these potentials, which was confirmed by control measurements carried out at three different Fe(II)/Fe(III) concentrations (Figure S17). Potentials of *V*_1_ < 0.0 V and *V*_2_ = 1.2 V vs RHE were applied to the Pt and Au electrodes, respectively, for studying the HER/HOR feedback in the supporting electrolyte.

To determine the influence of TiO_2_ coatings on the H_2_ oxidation back reaction, chronoamperometry scans in dual control were completed in supporting electrolyte with a similar set of Pt | Au electrodes, where the Au site was held at 1.2 V vs RHE while the Pt site was varied to potentials below 0 V vs RHE. As expected, there was minimal HOR occurring on the Au array even with large neighboring current densities, as Au is a poor HOR catalyst in acid (Figure S15).

Representative chronoamperometry scans of a bare Au | Pt sample in Fe-containing electrolyte are shown in Figure 7b, with scans for the AS-ALD and ALD electrodes available in Figure S16. Scans completed with potential control of only one of the electrode arrays (“single control”) exhibit pseudo-steady-state currents after 120 s of 0.12 and 0.14 mA cm^–2^ for the Au and Pt arrays, respectively. Dual control electrodes led to increases in the steady state currents of the bare Pt and Au arrays to 0.22 and 0.26 mA cm^–2^, respectively. These 83–85% increases in current when switching to dual control can be explained by positive feedback resulting from the generation of additional reactant species at neighboring band electrodes, as illustrated in Figure 7a. This observation highlights that the back reaction issue becomes amplified when the oxidation and reduction reaction sites are located close to each other. When reaction rates are limited by diffusion, as they are for the conditions employed here, the rates of back reactions due to positive feedback between neighboring sites can be expected to be amplified even further, as the distance between those sites gets even smaller than those used here.

The same measurements shown in Figure 7b for the uncoated interdigitated electrode sample were also carried out for 160 cycles of ALD and AS-ALD samples, with the average current densities recorded at 120 s for Pt and Au electrode arrays on these samples summarized in Figure 7c for both single and dual potential control. For the ALD sample containing TiO_2_ coating on both the Au and Pt electrodes, negligible FeOR and FeRR current is observed for either electrode under either potential control mode owing to the fact that the TiO_2_ coating blocks Fe(II)/Fe(III) redox chemistry as already shown in Figure 6c. In contrast, notable current densities were recorded for the AS-ALD sample for both single and dual potential control operations. The oxidation current measured for the Au electrodes on the AS-ALD sample was almost identical to that measured for the Au electrodes on the bare samples under single potential control. This result indicates that the AS-ALD process had minimal effect on the Au activity toward the FeOR. The reduction current for the Pt electrodes on the AS-ALD sample was significantly suppressed compared to the uncoated samples, but still exhibited a small current density of 0.04 and 0.05 mA cm^–2^ under single and dual control, respectively. This signal likely originates from FeRR on the outer surface of the TiO_2_ coating or through defects within the coating and/or reduction of oxygen from the ambient environment. More importantly, the increase in back reaction current was greatly diminished for the AS-ALD samples, even with the neighboring Au operating at a large FeOR current density.

The increases in FeRR and FeOR resulting from positive feedback between the Pt and Au arrays were estimated to be equal to the difference in the current measured using single and dual potential control with the results shown in Figure 7d. Importantly, there was a ∼ 10× decrease in feedback for the AS-ALD sample compared to the uncoated sample.

## Conclusions

This study developed an area-selective ALD process that can alter and define the selective reactivity of sites for a model hydrogen-evolving photocatalyst nanoreactor. In a dual-site catalyst system, modification of the surface chemistry of one catalyst without alteration of the neighboring catalyst creates a powerful tool in catalyst system design. Using interdigitated arrays of Pt and Au microelectrodes, this study demonstrated selective ALD of TiO_2_ on Pt while leaving Au uncoated. The nanoscopic oxide film was shown to selectively grow on Pt up to 160 ALD cycles, while additional cycles resulted in the nucleation of TiO_2_ on the Au. The selective deposition and electrochemical behavior were confirmed by XPS/STEM and SECM, respectively. Finally, the model interdigitated electrode platform was used to emulate neighboring reactive sites in a photocatalytic system that operates at different potentials. A key finding from this study was that interdigitated electrodes modified by AS-ALD exhibited a 10× decrease in undesired back reactions as compared to uncoated controls. This result highlights the promise of using AS-ALD to modify dual-site catalytic systems like particle-based photocatalysts to achieve higher reaction selectivity and consequently higher solar-to-fuel conversion efficiency. In future work, material processing procedures developed using the interdigitated array platform can guide the design and optimization of similar processes on nanoparticle systems, while catalyst properties and back reaction rates measured on this platform can be used by models that simulate the performance of ensembles of smaller scale structures.

## Methods and Materials

### Electrode Preparation

Monometallic electrodes were fabricated on degenerately doped p+ Si(100) wafers (Prime-grade p+Si, resistivity <0.005 Ω cm, 500–550 μm thick, WRS materials), with either 50 nm of Pt or Au electron beam deposited (Angstrom Ultra High Vacuum Nexdep) at a rate of 1 Å/s with a 2 nm Ti adhesion layer (deposited at 0.5 Å/s). Electrical contacts were attached by soldering a copper wire to the scratched back of the p+Si substrates using indium solder (Thermo Scientific, Puratronic, 99.999%) at a temperature of 220 °C. The geometric area of the electrode exposed to the electrolyte was defined using 3 M electroplaters tape with a circular opening of 0.25 cm^2^.

Octadecanethiol self-assembled monolayers were deposited by soaking electrodes overnight (∼16 h) in a 50 mM 1-octadecanethiol (Sigma-Aldrich, 98%) in ethanol (Fisher Scientific, reagent grade) solution. ALD TiO_2_ overlayers were deposited in a custom-made deposition reactor at 150 °C using alternating cycles of titanium(IV) chloride (TiCl_4_) and water in N_2_ carrier gas. One cycle consisted of a 1 s pulse of TiCl_4_, 1 s N_2_ purge, followed by a 1 s pulse of H_2_O and 3 s N_2_ purge. Samples with various cycles were synthesized all under continuous flow conditions.

Interdigitated Au and Pt electrodes were fabricated on either degenerately doped p-Si or glass substrates (Fisher Scientific, plain microscope slides) by first depositing 50 nm of the Au electrode features, followed by the ODT overnight soak and final deposition of 50 nm of Pt. Both Au and Pt layers included a 2 nm thick Ti adhesion layer. Interdigitated electrode features were defined by a custom pair of shadow masks (OSHstencils), which were mounted on a custom holder for alignment. Interdigitated electrodes on p-Si were deposited onto the nonpolished side to improve the adhesion of the TiO_2_ overlayer. Glass substrates were soaked overnight (∼16 h) in a 1 M sodium hydroxide (Certified ACS, Fisher Scientific) solution to improve metal (Pt, Au) adhesion. Top contacts to the interdigitated electrode contact pads were created with carbon paint (SPI, 05006-AB) and copper tape.

### Electrode Characterization

Thicknesses of the TiO_2_ coatings on monometallic electrodes were determined using an ellipsometer (J.A. Woollam α-SE) using angle scans from 70 to 80° with alignment at 75°. Thicknesses were determined by fitting the raw data to a Cauchy model with optical constants for TiO_2_. Fourier transform infrared (FTIR) spectra were performed using the attenuated total reflection (ATR) cell (Thermo Fischer Nicolet iS50). FTIR spectra were taken as the average of 64 scans with a resolution of 4, over a range of 4000–400 cm^–1^, gain 1.0, optical velocity 0.4747, and aperture of 10.

X-ray photoelectron spectroscopy measurements were made with a PHI Versaprobe 2 XPS system at pressures <2 × 10^–9^ Torr using a monochromatic Al Kα source (15 kV, 20 mA), tilted to 45° relative to the detector, and a charge neutralizer with samples electronically isolated from the stage. Multiplex spectra are shown as averages of four measurements, which were measured with a pass energy of 29.35 eV, a dwell time of 100 ms, and a beam size of 200 μm. XPS line scans were completed with a beam size of 20 μm, a pass energy of 117.4 eV, and a time per pixel of 20 ms. XPS area scans were completed with a beam size of 20 μm, a pass energy of 117.4 eV, and a time per pixel of 1 ms. Peaks were fitted using CasaXPS software applying Shirley’s algorithm for background subtraction. No additional shifts to the binding energy were applied during post-processing. Atomic ratios were calculated by normalizing the intensity of each element’s atomic sensitivity factor. Additional details on peak fitting procedures are provided in ESI Section II (Figures S2–S4).

Cross-sectional scanning transmission electron microscopy (STEM) and energy-dispersive spectroscopy (EDS) were performed using a JEOL Grand ARM 300CF microscope equipped with a cold field emission gun, double spherical aberration correctors, and double 100 mm^2^ X-ray detectors. This microscope operated at 300 kV, providing a spatial resolution of 63 pm. All HAADF-STEM images were acquired using a convergence semiangle of 21 mrad and inner- and outer-collection angles of 64 and 180 mrad, respectively. Cross-sectional lamellas were prepared using focused ion beam (FIB) milling (TESCAN GAIA-3 GMH integrated FIB-FESEM). The samples were thinned using successive milling by 30 kV, 8 kV, and 5 kV ion beams where a 2 kV beam was used for final cleaning.

### Electrochemical Measurements

Electrochemical measurements on monometallic planar electrodes were performed in deaerated aqueous 0.1 M Na_2_SO_4_ (ACS reagent, ≥ 99.0%, anhydrous, granular, Sigma-Aldrich) + 0.05 M H_2_SO_4_ (Certified ACS plus, Fischer Scientific) in 18 MΩ cm deionized water (Millipore, Milli-Q Direct 8) that was adjusted to pH 1.5 using concentrated sulfuric acid or sodium hydroxide. The Fe-containing electrolyte was prepared identically except for the addition of 25 mM FeSO_4_·7H_2_O (ACS reagent, ≥ 99.0%, Sigma-Aldrich) and 12.5 mM Fe_2_(SO_4_)_3_·×H_2_O (97%, Sigma-Aldrich). Electrolyte pH was measured with a benchtop pH meter (Fisher Science Education, S90526), using a 3-point calibration from 1.69, 4.01, and 7.00 standard buffers (Oakton). These electrochemical measurements were conducted with an SP-200 BioLogic potentiostat, a reversible hydrogen reference electrode (Hydroflex, ET070), a carbon rod (Saturn Industries, EDM3MINI12X.1100) counter electrode, and a three-neck round-bottom glass flask (Ace Glass, European flask, 250 mL). The electrolyte for all electrochemical experiments was deaerated by vigorously purging the electrolyte with nitrogen gas (Airgas, 99.99% purity) for 20 min prior to experimentation and blanketing the headspace of the cell with nitrogen throughout the subsequent experiments. Electrochemical testing in the supporting electrolyte (where applicable) was completed before any Fe-containing electrolyte.

The series resistance of each electrode was measured by performing potentiometric electrochemical impedance spectroscopy at open circuit potential from 200 kHz–100 mHz with an amplitude of 10 mV before cyclic voltammetry measurements. Pretreatment cyclic voltammetry measurements were performed between potentials of 0.05 and 1.15 V vs RHE with a scan rate of 100 mV s^–1^ for 20 cycles or until currents stabilized. Fe redox cyclic voltammetry measurements were performed between potentials of 0.3 and 1.2 V vs RHE for Pt-based electrodes and between 0 and 1.2 V vs RHE for Au-based electrodes with a scan rate of 10 mV s^–1^ for 2 cycles, and HER cyclic voltammetry scans were completed between potentials of −0.1 and 0.4 V vs RHE at 10 mV s^–1^ for 3 cycles.

Scanning electrochemical microscopy measurements were conducted using a CHI Instruments 920D bi-potentiostat and carried out in a custom-designed Teflon holder in 3 mM FeSO_4_ + 1.5 mM Fe_2_(SO_4_)_3_ + 0.1 M Na_2_SO_4_ + 0.05 M H_2_SO_4_ aqueous electrolyte. A commercial platinum 10 μm diameter SECM tip (CHI Instruments, CHI116) was used for all of the SECM measurements. A platinum wire was used as a pseudoreference electrode, where the potential stabilized near the reversible Fe(II)/Fe(III) potential and was calibrated to RHE based on the onset potential of the HER for the SECM tip. For supporting electrolyte scans, a commercial reversible hydrogen electrode was used as the reference electrode (Hydroflex, ET070). A carbon rod was used as the counter electrode. The electrolyte was prepurged with argon gas (Purity Plus 99.999% purity) for one hour before the start of measurements and blanketed over the cell during measurements. The SECM tip was positioned 25 μm above the surface of the electrode for all experiments, determined through approach curves (Figure S9). Measurement of feedback between neighboring interdigitated bands was performed in the SECM cell utilizing the bi-potentiostat to individually control the potentials of each pair of interdigitated electrodes.

## Supporting Information Available

Experimental methods and additional results (PDF)
