# Supplementary material for: Toward Spatial Control of Reaction Selectivity on Photocatalysts Using Area-Selective Atomic Layer Deposition on the Model Dual Site Electrocatalyst Platform
Source: ACS Nano. 2024 Dec 9;18(51):34708–19. doi: 10.1021/acsnano.4c10387 (PMC11673572; doi:10.1021/acsnano.4c10387)
Supplement: Supplementary file 1 — nn4c10387_si_001.pdf [file nn4c10387_si_001.pdf]

Electronic Supporting Information

Towards Spatial Control of Reaction Selectivity on Photocatalysts using Area Selective Atomic Layer Deposition on Model Dual Site Electrocatalyst Platform

W. Wilson McNeary†[a], William D. H. Stinson†[b], Moaz Waqar[c], Wenjie Zang[c], Xiaoqing Pan[c], Daniel V. Esposito*[b], Katherine E. Hurst*[d]

[a] Catalytic Carbon Transformation and Scale-up Center, National Renewable Energy Laboratory, Golden, CO 80401, USA

[b] Department of Chemical Engineering, Columbia Electrochemical Engineering Center, Lenfest Center for Sustainable Energy Columbia University in the City of New York, New York, NY 10027, USA

[c] Department of Materials Science and Engineering, University of California Irvine, Irvine, CA 92697, USA

[d] Energy Conversion and Storage Systems Center, National Renewable Energy Laboratory, Golden, CO 80401, USA

† indicates equal contribution

* de3200@columbia.edu; *katherine.hurst@nrel.gov

TABLE OF CONTENTS

SI. Additional FTIR ... 3
SII. XPS Analysis ... 4
SIII. Au monometallic cyclic voltammograms in supporting electrolyte ... 8
SIV. Additional STEM ... 9
SV. Additional XPS scans ... 11
SVI. SECM methodology ... 12
SVII. CVs of the Figure 6 (main article) substrates in SECM cell ... 15
SVIII. SECM imaging of area coating defect (160 cycles TiO_2 on AS-ALD) ... 16
SIX. Supporting electrolyte scan over AS-ALD interdigitated electrode ... 17
SX. Various cycle numbers on AS-ALD interdigitated ... 18
SXI. Additional feedback measurements ... 19
References ... 21

SI. Additional FTIR

The retention of ODT on the Au electrode surface following thermal annealing at the temperature used for ALD deposition (150 ˚C) was examined using FTIR (**Figure S1**). Under 100 sccm of He flow, the ODT-Au electrodes were heated in a tube furnace at 5 ˚C min^{-1} and held at the indicated temperature for 30 min. While thermal treatment was found to disrupt the ODT network and reduce the sharpness of the C-H stretching peaks at 2924 and 2853 nm^{-1} (which were clearly not present on bare Au), the continued existence of the C-H peaks after annealing confirms that the ODT should still be present during the TiO_2 AS-ALD process at 150 ˚C.

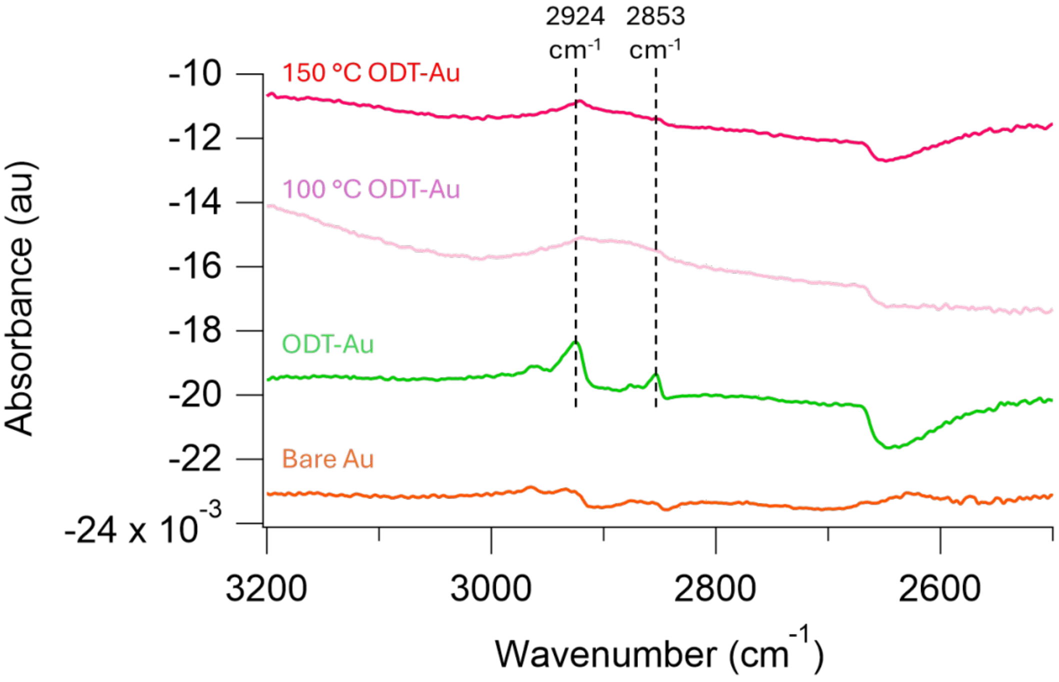

Figure S1: FTIR spectra of monometallic bare Au (orange trace), ODT-Au as-made (green trace), after 100 ˚C anneal (pink trace), and after 150 ˚C anneal (red trace) in a Helium environment.

SII. XPS Analysis

X-ray photoelectron spectroscopy (XPS) measurements were made using a PHI Versaprobe 2 XPS system at pressures < 2 × 10^{-9} Torr using a monochromatic Al Kα source (15 kV, 20 mA), tilted to 45° relative to the detector, and a charge neutralizer with samples electronically isolated from the stage. Multiplex spectra are shown as averages of four measurements with a pass energy of 29.35 eV and 100 ms dwell time using a spot size of 200 μm. Scans were fitted using CasaXPS software, with a Shirley model used to background all spectra. No additional shifts to the binding energy were applied during post processing. Additional O1s and C1s spectra of the electrodes in Section 2.1 (main article) and the full multiplex spectra for Pt electrodes can be seen in **Figure S2**, and **Figure S3**, respectively.

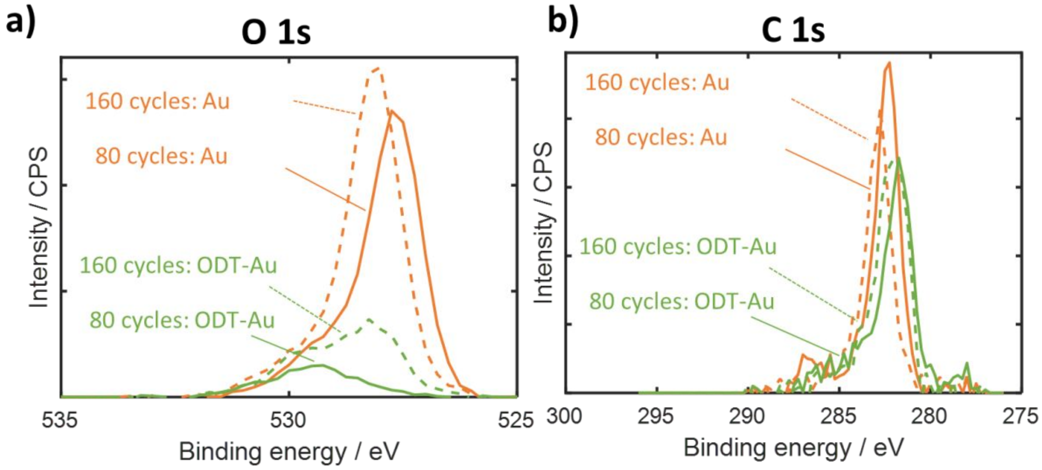

Figure S2: XPS of the **a)** O 1s and **b)** C 1s regions of electrodes seen in Figure 2 (main text), with Au (orange trace) and ODT-Au (green trace) surfaces subjected to 80 (solid trace) and 160 (dotted trace) ALD cycles of TiO_2.

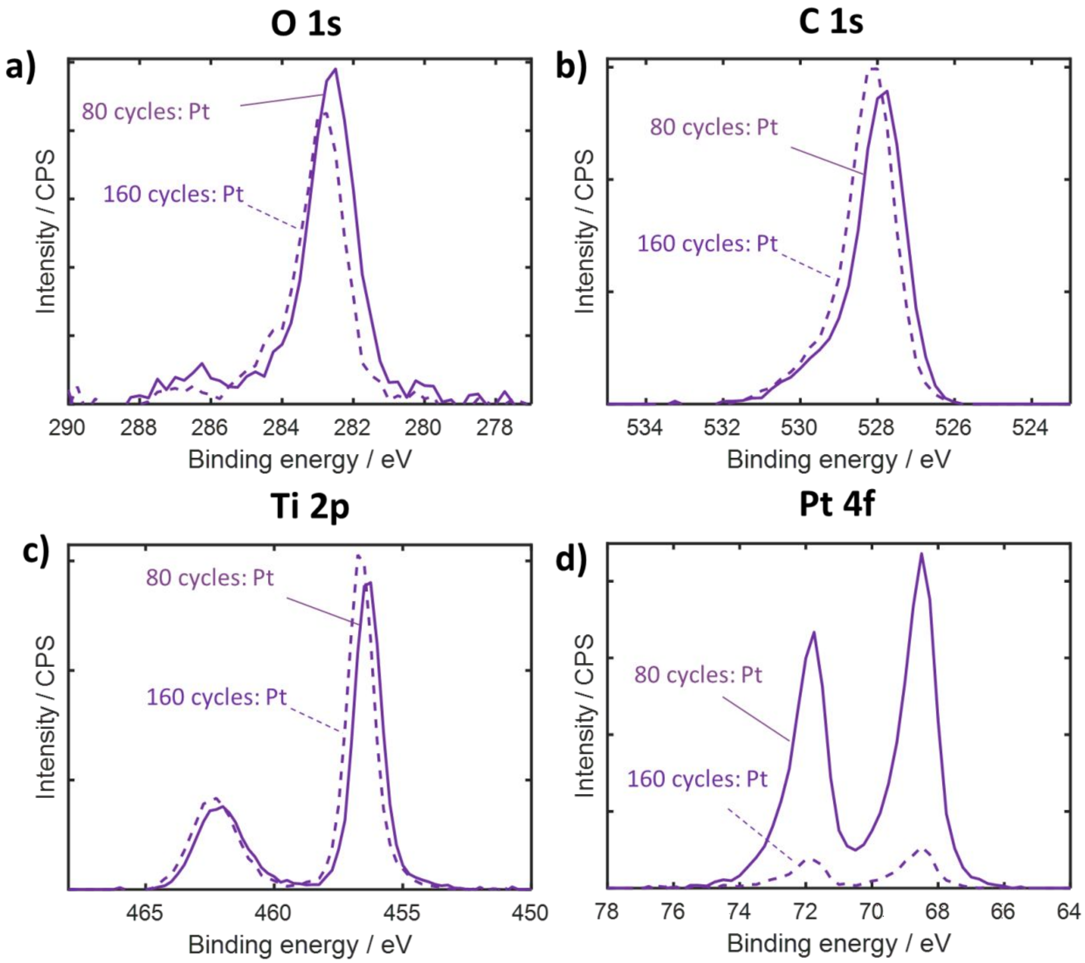

Figure S3: XPS of the **a)** O 1s, **b)** C 1s, **c)** Ti 2p, and **d)** Pt 4f regions of Pt electrodes subjected to 80 (solid trace) and 160 (dotted trace) ALD cycles of TiO_2.

To determine the Ti:O ratio, the O 1s and Ti 2p regions were fitted with a Gaussian-Lorentzian (GL) line shape, with 70% Gaussian behavior, and constrained to have full width at half maximum (FWHM) to be < 2. Ti 2p spectra were fitted with a doublet with 1/2-3/2 peak splitting of 5.72 eV, where it was found to have a slight difference in peak binding energy (~ 456 eV) as compared to literature values (~ 458 eV).[1,2] O 1s peaks were fitted with two GL peaks, associated with lattice (Ti-O-Ti, ~ 528 eV) and surface oxygen (Ti-OH or adsorbed water, ~529.5 eV). The difference between these two peaks (1.3-1.5 eV) was found to be similar to those found in literature (1.5-1.8 eV) for electrodes where TiO_2 is expected to grow (Au, Pt).[3] ODT modified Au was found to have minimal Ti 2p signal at 80 cycles, and appreciable signal at 160 cycles. The presence of a larger surface O peak for the 160 cycles ODT-Au suggests island growth, where a higher surface to volume ratios would be expected. The 80 cycles ODT-Au displays a significant O 1s peak, which may be related to a small amount of TiO_2 growth but could also originate from adventitious oxygen-containing carbon species, absorbed H_2O, or oxidized Au originating from

the deposition process. Since the origin was unknown, it was fitted with a singular broad (FWHM ~2.5) O 1s peak. The ratio of the Ti 2p peaks and the O 1s, associated with lattice oxygen, was found to be 1.78-1.88 when normalized using the intensity of each element's atomic sensitivity factor.[4] Fitted Ti 2p and O 1s peaks can be seen in **Figure S4** for all electrodes. **Table S1** lists peak locations, O 1s and Ti:O ratios of all Au, Pt, and ODT-Au samples.

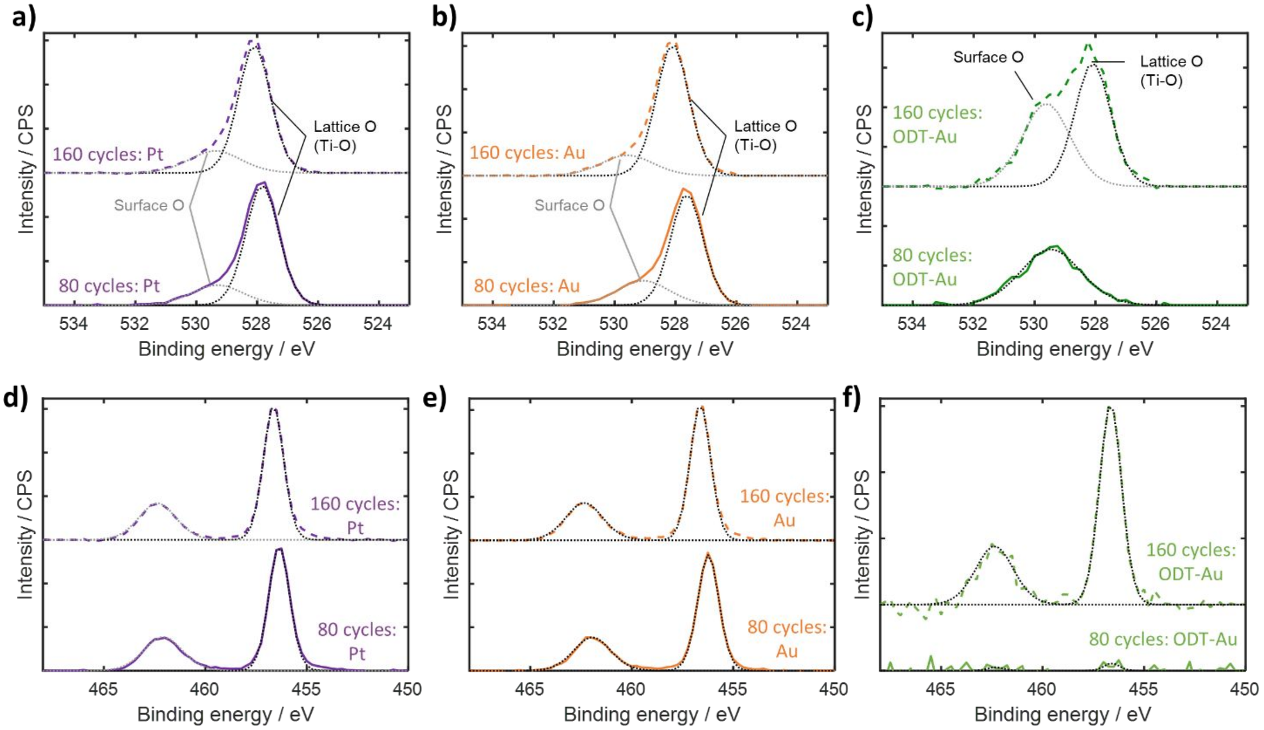

Figure S4: XPS **(a-c)** O1s regions and **(d-f)** Ti 2p regions with peak fitting for **(a,d)** Pt, **(b,e)** Au and **(c,f)** ODT-Au electrodes. Peaks were fitted with 70% Gaussian behavior on GL peaks, with FWHM constrained to < 2.

Table S1: Peak fitting locations and ratios of all ALD coated electrodes

Sample	Ti 2p 3/2 peak location / eV	O1s peak location / eV	O1s (lattice) to O1s (surface) ratio	O 1s to Ti 2p ratio
80 cycles: Pt	456.3	527.6 / 529.0	3.8	1.86
160 cycles: Pt	456.8	528.1 / 529.4	3.6	1.78
80 cycles: Au	456.3	527.6 / 529.0	2.8	1.82
160 cycles: Au	456.5	528.1 / 529.6	4.0	1.86
80 cycles: ODT-Au	n/a	529.5	n/a	n/a
160 cycles: ODT-Au	456.8	528.1 / 529.6	1.1	2.18

SIII. Au monometallic cyclic voltammograms in supporting electrolyte

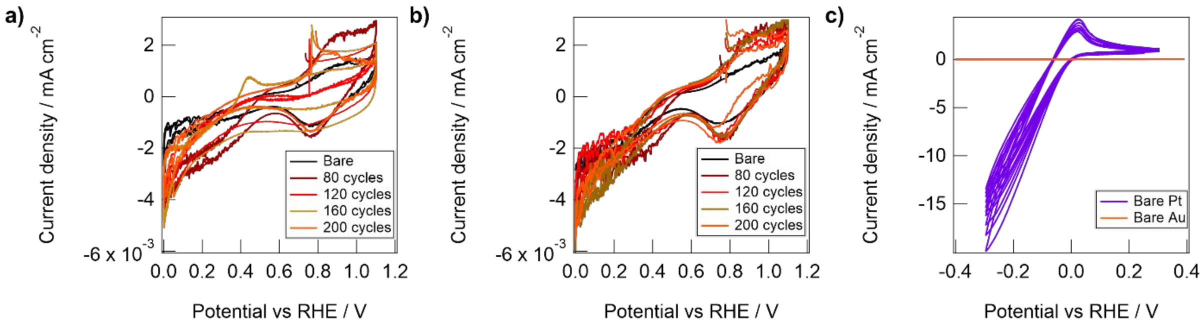

Figure S5: Cyclic voltammograms (CV) of **a)** Au and **b)** ODT-Au **c)** extended scan range bare Pt and bare Au electrodes in N_2-purged supporting electrolyte (50 mM H_2SO4 + 100 mM Na_2SO_4, pH 1.5), taken at 10 mV/s.

SIV. Additional STEM

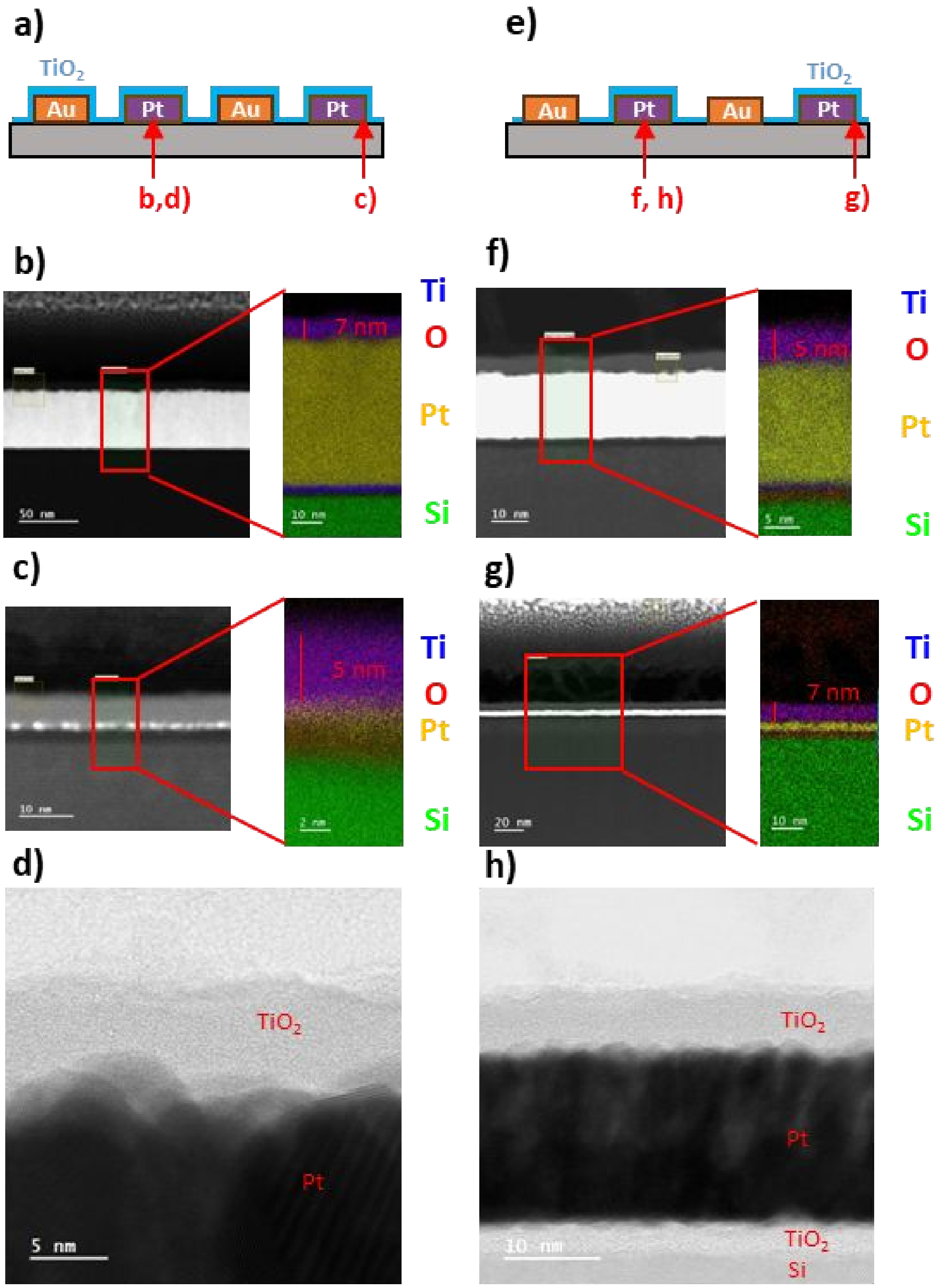

Figure S6. Scanning transmission microscopy images/elemental mapping (Au, O, Ti, Si) on Pt features of **(a, b, c, d)** 160 cycles TiO_2 on Pt | Au electrode and **(e, f, g, h)** 160 cycles TiO_2 on Pt | ODT-Au electrode. Panels **d)** and **h)** display high-magnification images over the center region of the denoted Pt bands. A schematic of the cross-sectional samples is included to help visualize locations of metallic band electrodes in **a** and **e**, as well as TEM locations in **(b-h)**.

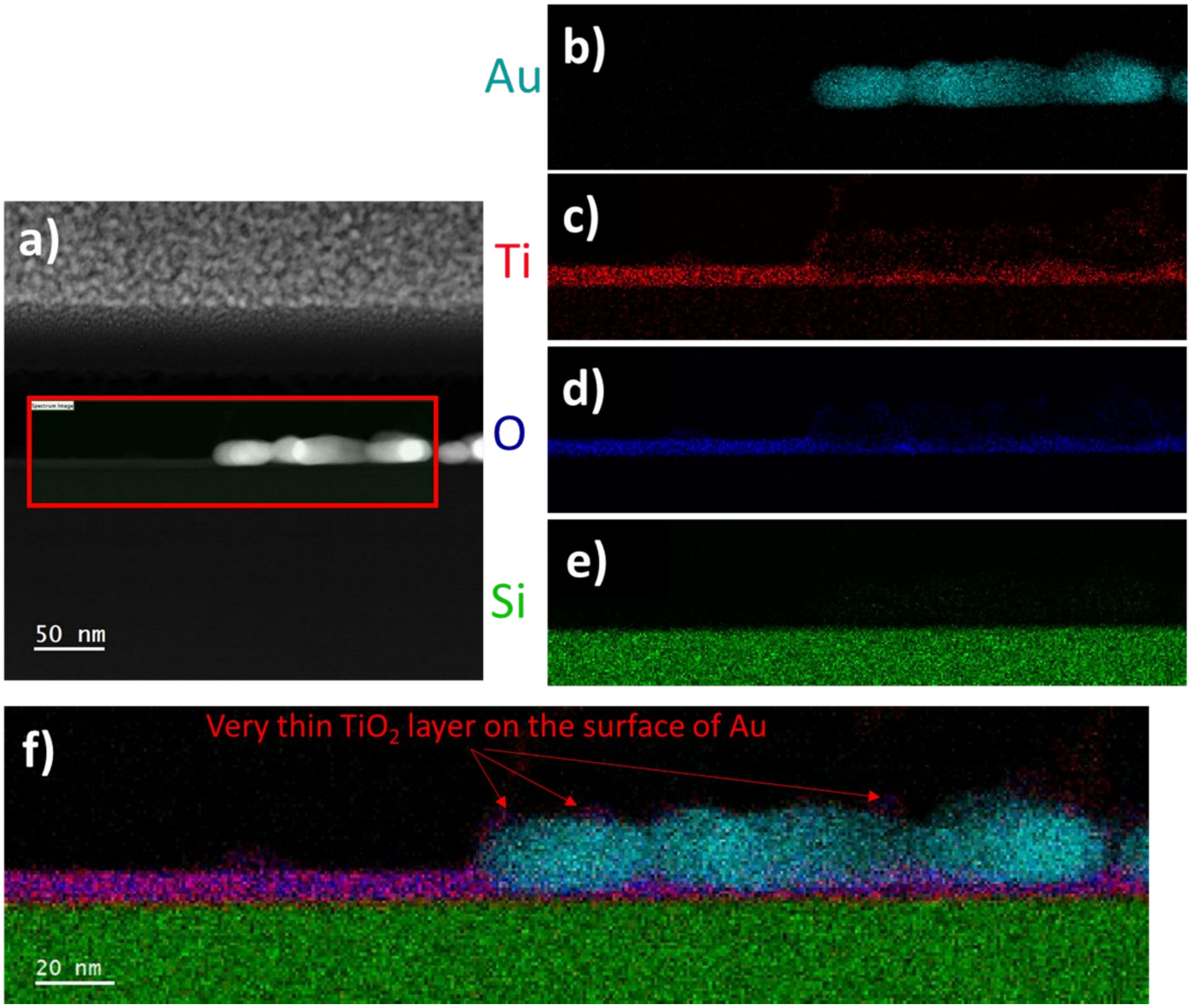

Figure S7: **a)** TEM image of Au edge region of 160 cycles TiO_2 AS-ALD sample with EDX of **b)** Au, **c)** Ti, **d)** O, and **e)** Si, with **f)** overlay of EDX showcasing trace TiO_2 on the surface of Au. Panels **b)-f)** are higher-resolution images corresponding to the area indicated by the rectangle in **a)**.

SV. Additional XPS scans

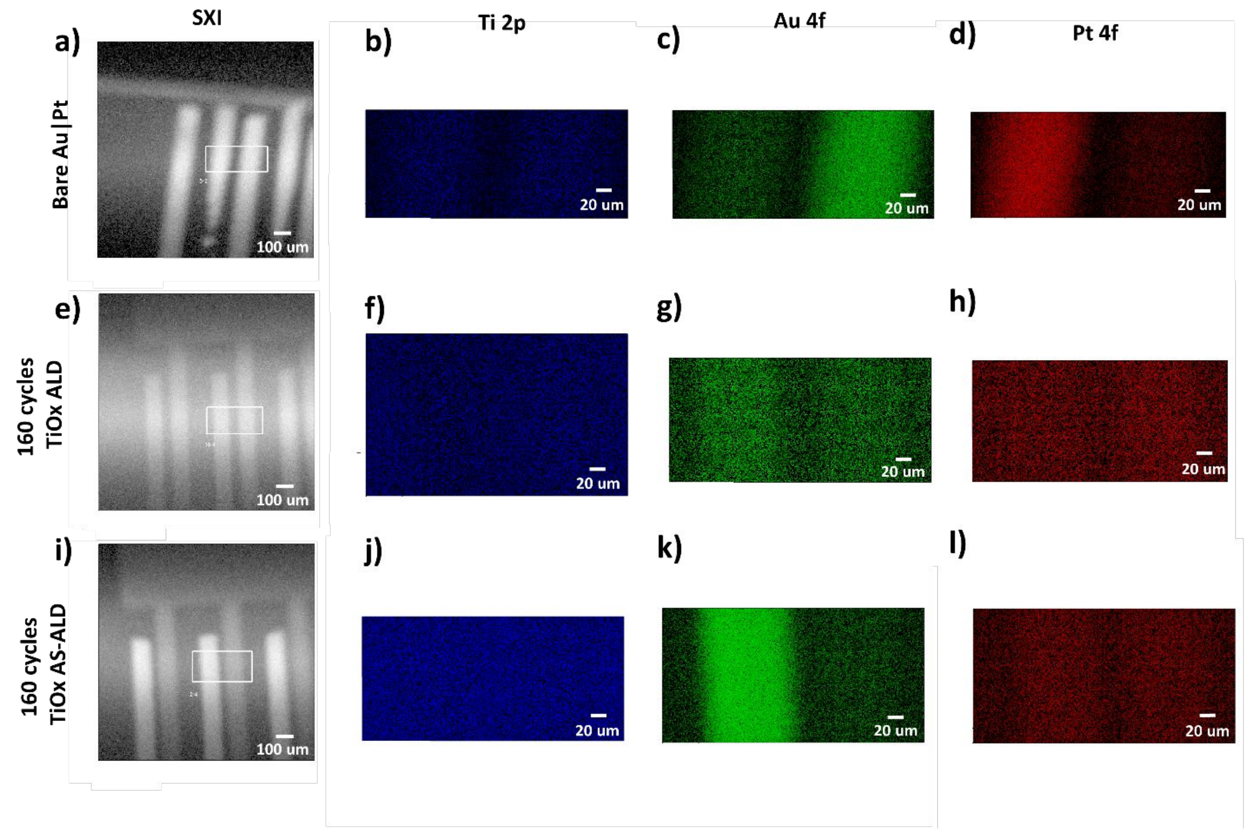

Figure S8: XPS images recorded over the regions defined by the white rectangles superimposed on the **(a,e,i)** secondary X-ray images of bare, ALD and AS-ALD interdigitated band electrodes. XPS images are provided based on the **(b,f,j)** Ti 2p,**(c,g,k)** Au 4f and **(d,h,l)** Pt 4f regions.

SVI. SECM methodology

SECM measurements were completed on three types of interdigitated band electrodes: uncoated, ALD, and AS-ALD interdigitated electrodes. Samples were mounted in a custom machined Teflon holder, with 3M Electroplater's tape used to define the exposed area of each sample. An example cyclic voltammogram of a 10 µm diameter UME tip can be seen in **Figure S9a**, which was measured in Fe containing electrolyte (3 mM FeSO4 + 1.5mM $Fe_2(SO_4)_3$ + 50 mM H_2SO_4 + 100mM Na_2SO_4). Substrate-tip separation distance was fixed at 25 µm measured through approach curves (**Figure S9b**), where SECM feedback is reduced while minimizing diffusional distance. Approach curves were completed with a tip potential of 0.1 V vs RHE and scan speed of 3 µm s^{-1}. The x-y location of the probe with respect to features on the sample surface was first determined through a 2D SECM scan of the corner region of the sample (**Figure S9d**), for which an optical image is also shown in **Figure S9c**. SECM images were completed with tip potential held at 0.1 V vs RHE and scanned over a 1000 um by 1000 um region at 100 um s^{-1}.

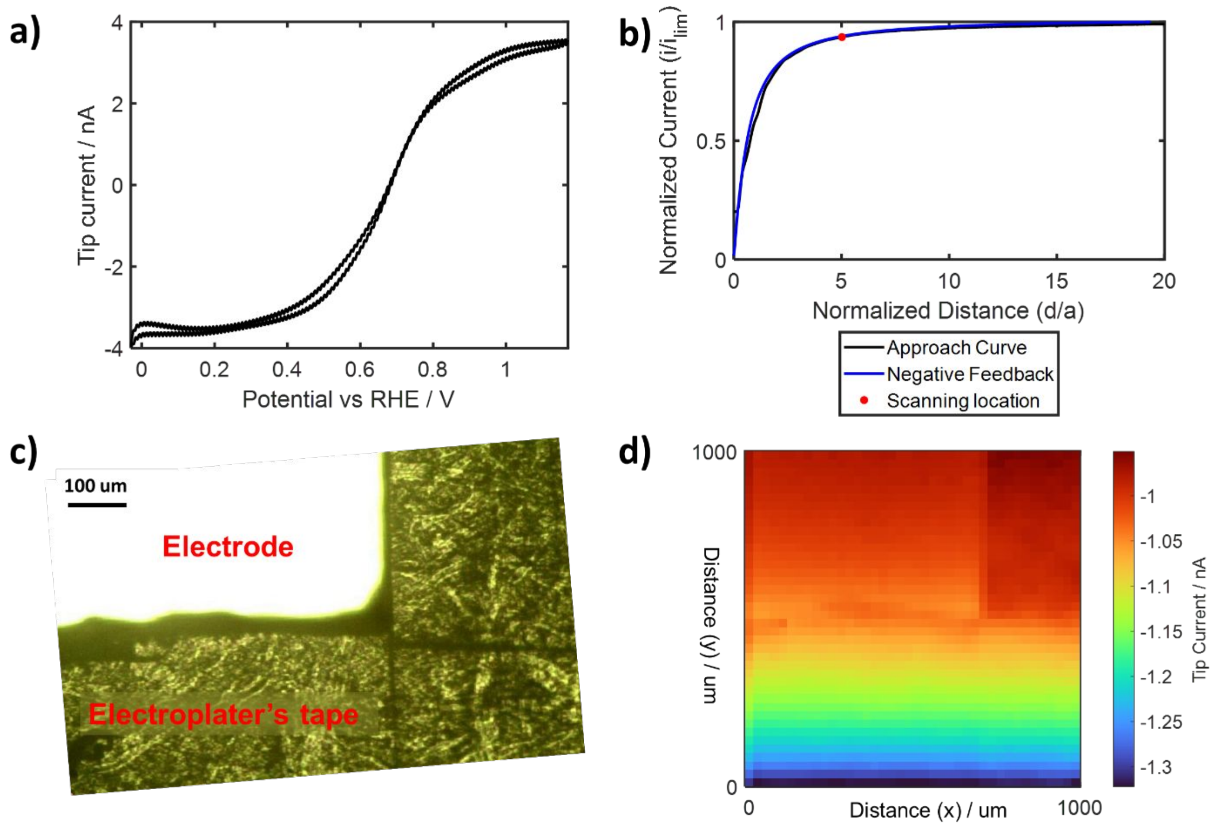

Figure S9: **a)** Cyclic voltammogram of 10 um diameter Pt UME in Fe containing electrolyte (3 mM FeSO4 + 1.5mM $Fe_2(SO_4)_3$ + 50 mM H_2SO_4 + 100mM Na_2SO_4) at scan speed of 100 mV s^{-1}. **b)** Scanning electrochemical approach curve over non-metallic interdigitated electrode with tip potential of 0.1 V vs RHE in Fe containing electrolyte, where distance away from substrate (d)

was normalized with tip radius (a). Negative feedback was modeled using methodology described by Bard and Mirkin,[5] with SECM scanning distance highlighted at 25 μm (red marker). **c)** Optical micrograph of the electroplater's tape corner acting as a fiducial marker with **d)** SECM image recorded over the same region as **c)** with a tip potential of 0.1 V vs RHE.

SECM linescans were used to estimate the partial currents towards each reaction over each metal band electrode. To do so, the tip was held at a constant potential and two subsequent scans were completed at varying potentials, with the first measuring the background current signal and the second estimating the reaction of interest. This methodology, similar to that described in in Stinson et al.,[6] was completed with the potentials listed below in **Table S2** for each reaction. Estimation of HER partial current was completed with a tip potential around reversible Fe(II)/Fe(III) potential to limit background current associated with the Fe redox reactions, while the FeRR estimate was completed with a tip potential where the Fe(III) was under mass transport limitation. Exemplary line scans at each potential are seen in **Figure S9** for all three electrodes.

Table S2: Potentials used for SECM experiments

**Partial current estimated**	**Tip Potential**	**Substrate potentials**
FeRR	0.1 V vs RHE	0.1 V vs RHE (measure $Fe^{(III)}$)<br>Open circuit potential (Background)
HER	0.8 V vs RHE	-0.05 V vs RHE (measure H_2)<br>0.1 V vs RHE (Background)
HER (Static tip CV)	0.8 V vs RHE	CV between [-0.1, 1.2V] vs RHE at 20 mV s^{-1}

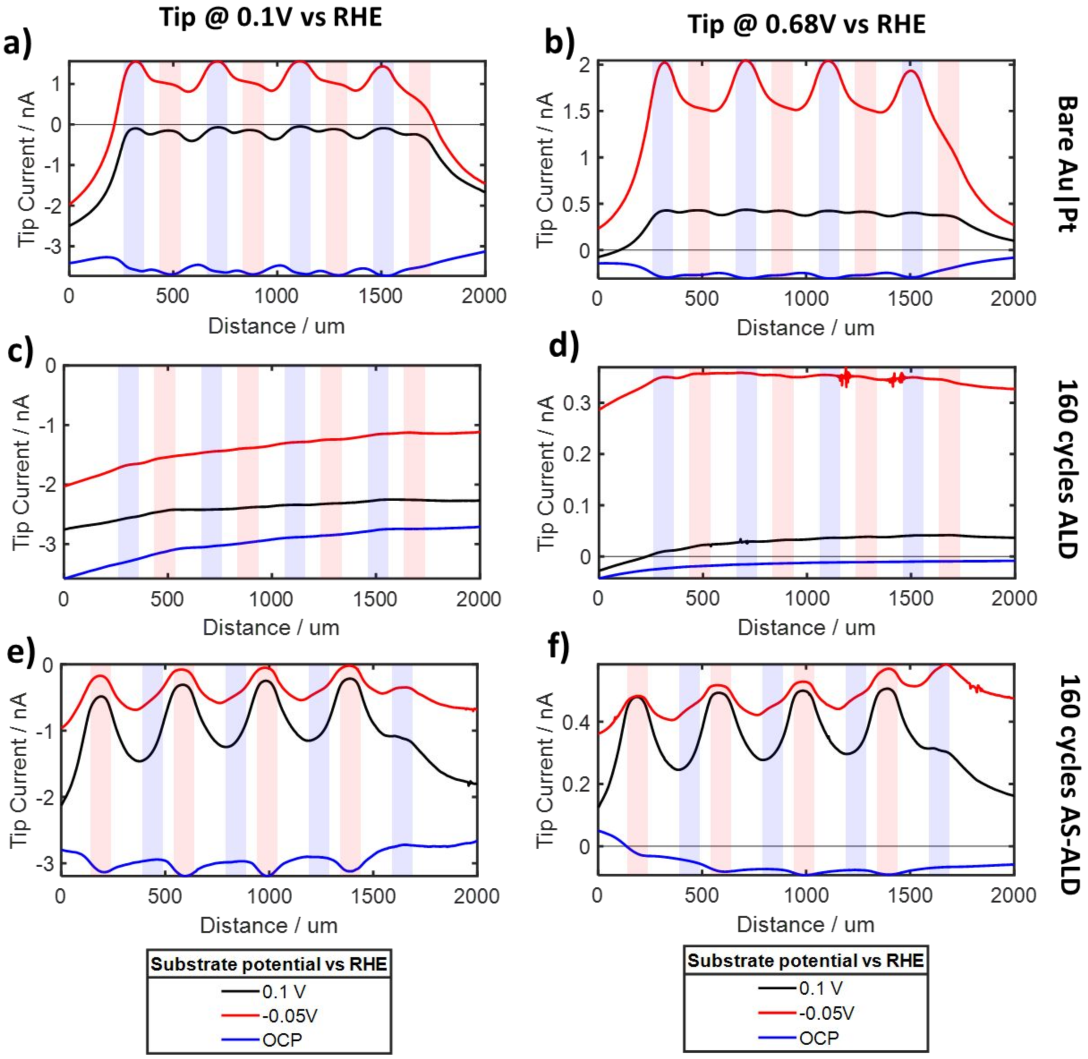

Figure S10: SECM line scans over **(a,b)** bare **(c,d)** ALD and **(e,f)** AS-ALD Au|Pt interdigitated electrodes seen in **Figure 5** in the main text. Scans were completed at three different substrate potentials and two different tip potentials of **(a,c,e)** 0.1V vs RHE and **(b,d,f)** 0.68 V vs RHE in Fe containing electrolyte (100 mM Na_2SO_4 + 50mM H_2SO_4 + 3 mM $FeSO_4$ + 1.5 mM $Fe_2(SO_4)_3$, pH 1.5).

SVII. CVs of the Figure 6 (main article) substrates in SECM cell

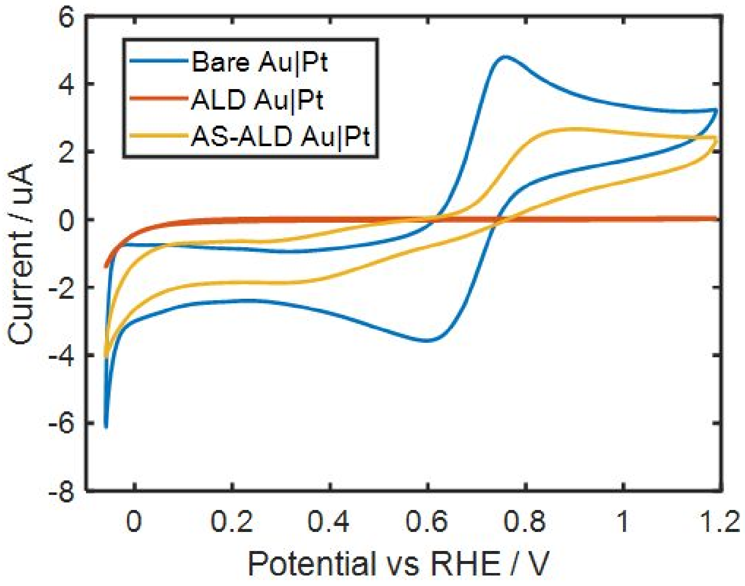

Figure S11: Cyclic voltammograms of an uncoated and 160 cycles ALD, and 160 cycles AS-ALD AulPt interdigitated band electrodes at scan speed of 100 mV s^{-1} in Fe containing electrolyte (100 mM Na_2SO_4 + 50mM H_2SO_4 + 3 mM $FeSO_4$ + 1.5 mM $Fe_2(SO_4)_3$, pH 1.5).

SVIII. SECM imaging of area coating defect (160 cycles TiO_2 on AS-ALD)

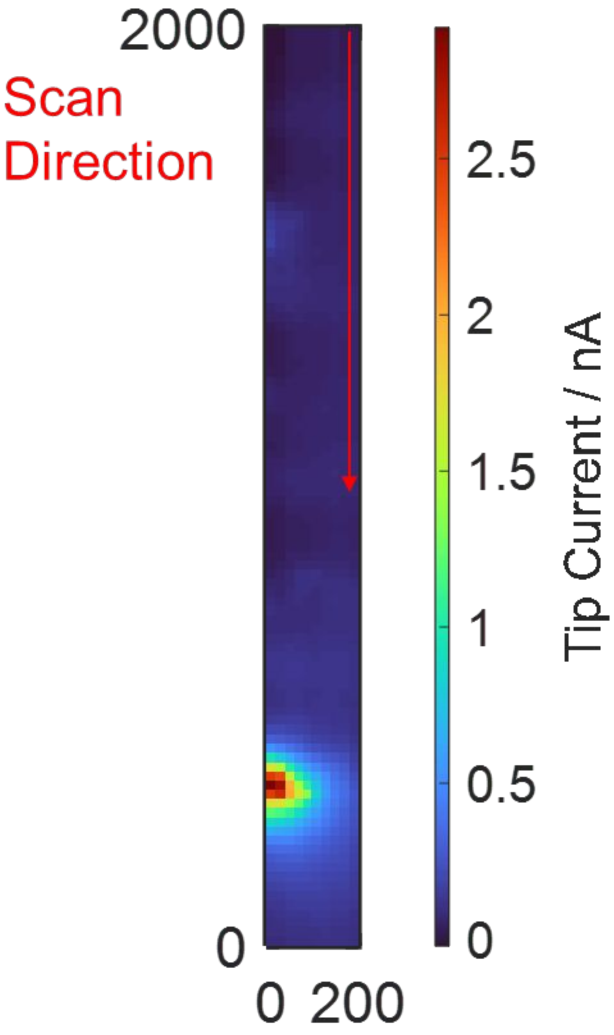

Figure S12. SECM area scan over interdigitated 160 cycle TiO_2 AS-ALD electrode. SECM scan was completed with substrate potential of -0.05 V vs RHE and tip potential of 0.1 V vs RHE at 100 um s^{-1} in Fe containing electrolyte (100 mM Na_2SO_4 + 50mM H_2SO_4 + 3 mM $FeSO_4$ + 1.5 mM $Fe_2(SO_4)_3$, pH 1.5).

SIX. Supporting electrolyte scan over AS-ALD interdigitated electrode

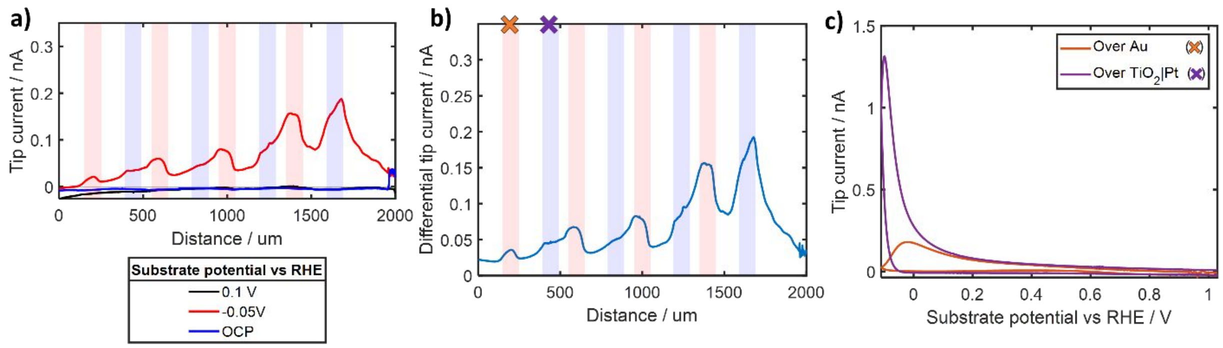

Figure S13: **a)** SECM line scans over 160 cycle TiO_2 AS-ALD interdigitated electrode in supporting electrolyte (50 mM H_2SO_4 + 100M Na_2SO_4, pH 1.5) with a tip potential of 0.75 V vs RHE. **b)** Estimated partial current towards HER, taken as the difference between line scans at substrate potentials of -0.05 V and 0.1 V vs RHE. **c)** Tip current measured with a tip potential of 0.75 V vs RHE while located over AS-ALD sample at marked (X) locations in **(b)** while the substrate was cycled between -0.1 V and 1.1 V vs RHE at 20 mV s^{-1}.

SX. Various cycle numbers on AS-ALD interdigitated

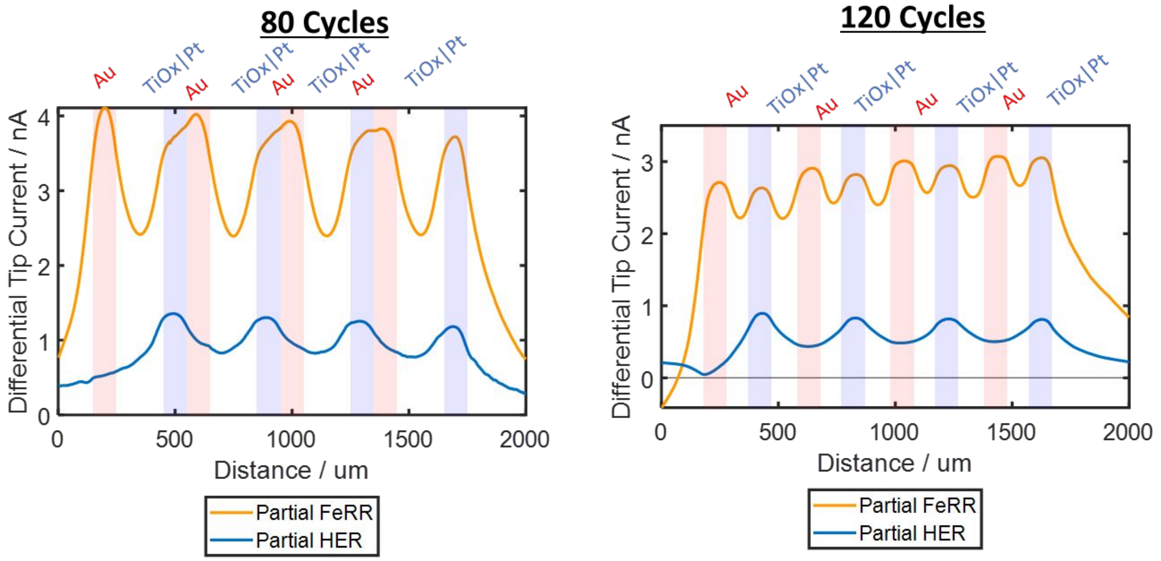

Figure S14: Estimated partial currents measured by scanning a UME perpendicular to the length of the interdigitated electrodes for the FeRR and the HER of **a)** 80 cycles and, **b)** 120 cycles TiO_2 AS-ALD interdigitated electrodes in Fe containing electrolyte (100 mM Na_2SO_4 + 50mM H_2SO_4 + 3 mM $FeSO_4$ + 1.5 mM $Fe_2(SO_4)_3$, pH 1.5).

SXI. Additional feedback measurements

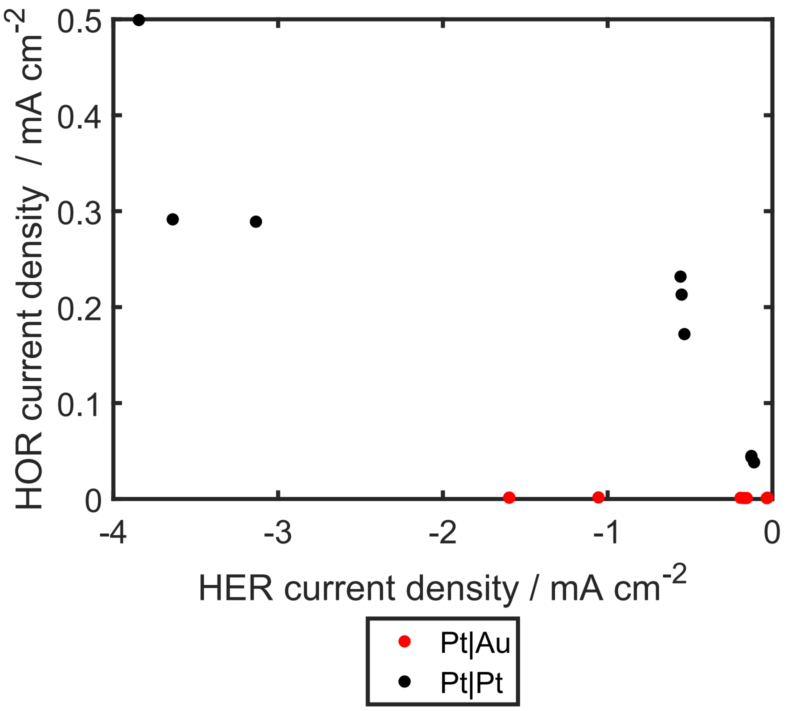

Figure S15: Extracted steady state currents of bare interdigitated electrodes in supporting electrolyte with independent control (Au at 1.2 V vs RHE, Pt at [-0.01, 0.1 V] vs RHE) and both under applied potential at varying Pt potentials. Minimal oxidation current seen on Au substrate suggests HOR is limited on Au features.

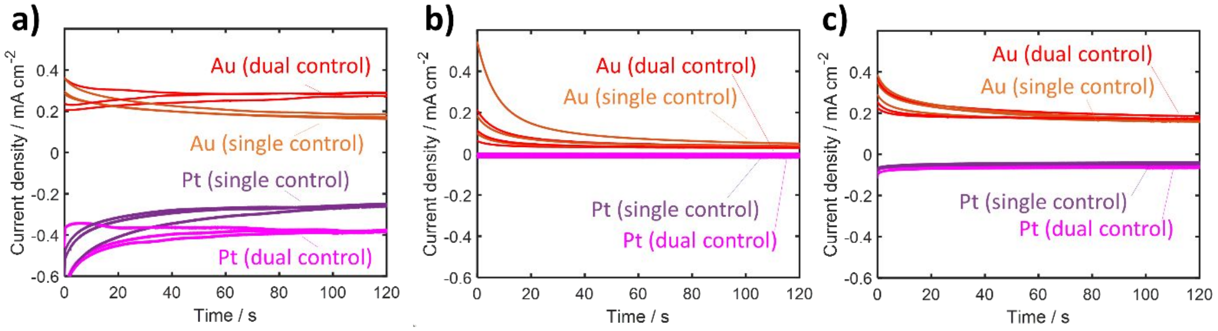

Figure S16: Chronoamperometry measurements of **a)** bare, **b)** ALD and **c)** AS-ALD electrodes with independent control (Au at 1.2 V vs RHE, Pt at 0.1 V vs RHE) and both under applied potential in Fe containing electrolyte (100 mM Na_2SO_4 + 50mM H_2SO_4 + 25 mM $FeSO_4$ + 12.5 mM $Fe_2(SO_4)_3$, pH 1.5).

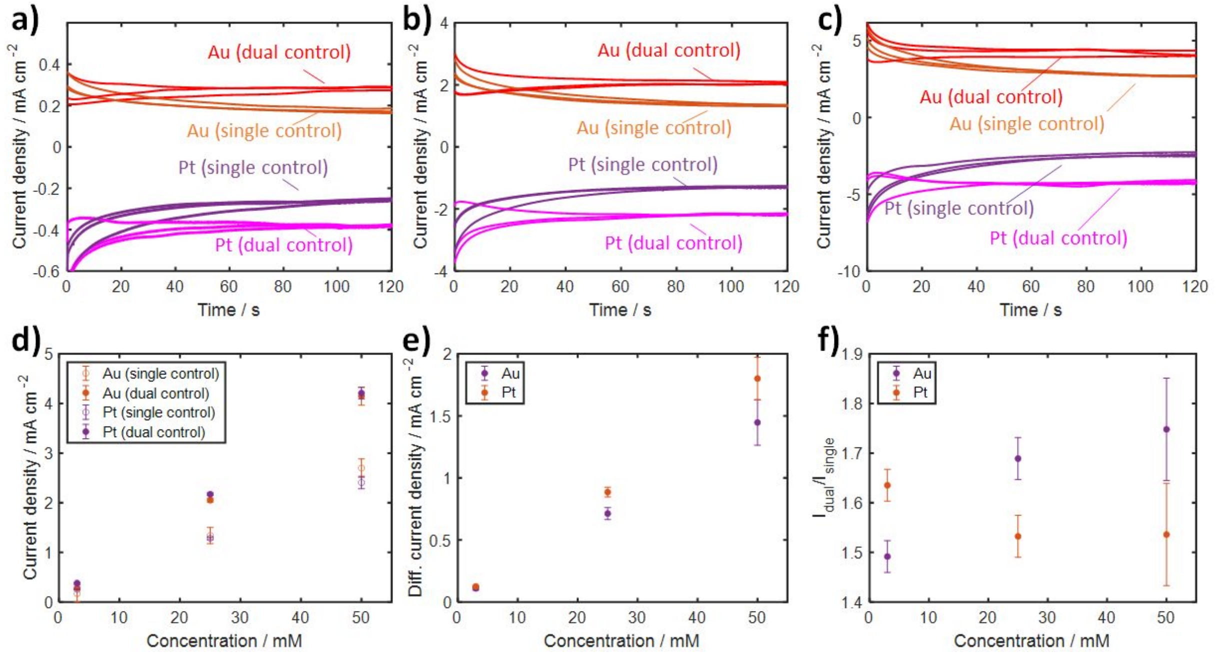

Figure S17: Chronoamperometry measurements of bare interdigitated electrodes at **a)** 3mM, **b)** 25 mM, and **c)** 50 mM Fe(II)/Fe(III) with independent control (Au at 1.2 V vs RHE, Pt at 0.1 V vs RHE) and both under applied potential. **d)** Extracted steady state current at the three Fe concentrations, taken as average over 110-120 s. **e)** Differential current density between the single and dual potential control plotted as a function of the Fe concentration, and **f)** ratio of the two potential controls. Linear slopes demonstrate that feedback currents are dependent on Fe concentrations, and thus controlled by diffusion between neighboring electrodes.

References

(1) Biesinger, M. C.; Lau, L. W. M.; Gerson, A. R.; Smart, R. St. C. Resolving Surface Chemical States in XPS Analysis of First Row Transition Metals, Oxides and Hydroxides: Sc, Ti, V, Cu and Zn. *Applied Surface Science* **2010**, *257* (3), 887–898. https://doi.org/10.1016/j.apsusc.2010.07.086.

(2) Finke, C. E.; Omelchenko, S. T.; Jasper, J. T.; Lichterman, M. F.; Read, C. G.; Lewis, N. S.; Hoffmann, M. R. Enhancing the Activity of Oxygen-Evolution and Chlorine-Evolution Electrocatalysts by Atomic Layer Deposition of TiO2. *Energy Environ. Sci.* **2019**, *12* (1), 358–365. https://doi.org/10.1039/C8EE02351D.

(3) Simmons, G. W.; Beard, B. C. Characterization of Acid-Base Properties of the Hydrated Oxides on Iron and Titanium Metal Surfaces. *J. Phys. Chem.* **1987**, *91* (5), 1143–1148. https://doi.org/10.1021/j100289a025.

(4) Briggs, D. Handbook of X-Ray Photoelectron Spectroscopy C. D. Wanger, W. M. Riggs, L. E. Davis, J. F. Moulder and G. E.Muilenberg Perkin-Elmer Corp., Physical Electronics Division, Eden Prairie, Minnesota, USA, 1979. 190 Pp. *Surface and Interface Analysis* **1981**, *3* (4), v–v. https://doi.org/10.1002/sia.740030412.

(5) *Scanning Electrochemical Microscopy*, 2nd ed.; Bard, A. J., Mirkin, M. V., Eds.; CRC Press: Boca Raton, 2012. https://doi.org/10.1201/b11850.

(6) Stinson, W. D. H.; Brayton, K. M.; Ardo, S.; Talin, A. A.; Esposito, D. V. Quantifying the Influence of Defects on Selectivity of Electrodes Encapsulated by Nanoscopic Silicon Oxide Overlayers. *ACS Appl. Mater. Interfaces* **2022**, *14* (50), 55480–55490. https://doi.org/10.1021/acsami.2c13646.
